# Microbial Metabolism Shifts Towards an Adverse Profile with Supplementary Iron in the TIM-2 *In vitro* Model of the Human Colon

**DOI:** 10.3389/fmicb.2015.01481

**Published:** 2016-01-06

**Authors:** Guus A. M. Kortman, Bas E. Dutilh, Annet J. H. Maathuis, Udo F. Engelke, Jos Boekhorst, Kevin P. Keegan, Fiona G. G. Nielsen, Jason Betley, Jacqueline C. Weir, Zoya Kingsbury, Leo A. J. Kluijtmans, Dorine W. Swinkels, Koen Venema, Harold Tjalsma

**Affiliations:** ^1^Department of Laboratory Medicine – Translational Metabolic Laboratory, Radboud Institute for Molecular Life Sciences, Radboud University Medical CenterNijmegen, Netherlands; ^2^Radboud Institute for Molecular Life Sciences, Center for Molecular and Biomolecular Informatics, Radboud University Medical CenterNijmegen, Netherlands; ^3^Theoretical Biology and Bioinformatics, Utrecht UniversityUtrecht, Netherlands; ^4^Department of Marine Biology, Institute of Biology, Federal University of Rio de JaneiroRio de Janeiro, Brazil; ^5^TNO Innovation for LifeZeist, Netherlands; ^6^NIZO food research, Kernhemseweg EdeNetherlands; ^7^Argonne National Laboratory, University of ChicagoLemont, IL, USA; ^8^Illumina Inc., Chesterford Research ParkLittle Chesterford, UK; ^9^DNAdigest, Future Business CentreCambridge, UK

**Keywords:** *in vitro* fermentation, iron supplementation, gut microbiome, metabolome, metagenome, toxicity, SCFA, BCFA

## Abstract

Oral iron administration in African children can increase the risk for infections. However, it remains unclear to what extent supplementary iron affects the intestinal microbiome. We here explored the impact of iron preparations on microbial growth and metabolism in the well-controlled TNO's *in vitro* model of the large intestine (TIM-2). The model was inoculated with a human microbiota, without supplementary iron, or with 50 or 250 μmol/L ferrous sulfate, 50 or 250 μmol/L ferric citrate, or 50 μmol/L hemin. High resolution responses of the microbiota were examined by 16S rDNA pyrosequencing, microarray analysis, and metagenomic sequencing. The metabolome was assessed by fatty acid quantification, gas chromatography-mass spectrometry (GC-MS), and ^1^H-NMR spectroscopy. Cultured intestinal epithelial Caco-2 cells were used to assess fecal water toxicity. Microbiome analysis showed, among others, that supplementary iron induced decreased levels of *Bifidobacteriaceae* and *Lactobacillaceae*, while it caused higher levels of *Roseburia* and *Prevotella*. Metagenomic analyses showed an enrichment of microbial motility-chemotaxis systems, while the metabolome markedly changed from a saccharolytic to a proteolytic profile in response to iron. Branched chain fatty acids and ammonia levels increased significantly, in particular with ferrous sulfate. Importantly, the metabolite-containing effluent from iron-rich conditions showed increased cytotoxicity to Caco-2 cells. Our explorations indicate that in the absence of host influences, iron induces a more hostile environment characterized by a reduction of microbes that are generally beneficial, and increased levels of bacterial metabolites that can impair the barrier function of a cultured intestinal epithelial monolayer.

## Introduction

Iron deficiency is the most prevalent nutritional disorder worldwide, mostly affecting infants, young children, and women in developing countries. This deficiency has major health consequences such as poor pregnancy outcome, and impaired physical and cognitive development of children (WHO and UNICEF, 2004; Muthayya et al., 2013). Several trials have shown that iron deficiency can be effectively controlled by oral iron administration (Zimmermann and Hurrell, 2007). However, the uptake of iron from the intestines is influenced by many factors and is usually limited (Hurrell and Egli, 2010). Iron supplementation therefore generally results in a large fraction of unabsorbed iron entering the colon, being potentially available for the gut microbiota.

Importantly, oral iron administration is known to increase the incidence of diarrhea and has been associated with increased gut inflammation (Gera and Sachdev, 2002; Zimmermann et al., 2010; Jaeggi et al., 2014). It is therefore highly warranted to investigate the effects of nutritional iron on the gut microbiota composition and metabolic activity. We previously showed that iron can enhance the growth and virulence of gut bacterial pathogens in pure cultures (Kortman et al., 2012). Furthermore, it was recently shown that iron fortification caused a potentially more pathogenic gut microbiota profile (i.e., increased relative abundance of *Enterobacteriaceae*, including pathogenic *Escherichia coli*, and a decrease of generally beneficial *Lactobacillaceae* and/or *Bifidobacteriaceae*) in African children and infants (Zimmermann et al., 2010; Jaeggi et al., 2014). This increase in the abundance of fecal *Enterobacteriaceae* correlated with an increase of fecal calprotectin (Zimmermann et al., 2010), which is a marker of gut inflammation. Notably, the reported effect of oral iron administration on the gut microbiota composition varies between studies. The most consistent outcome of iron supplementation appears to be a decrease in *Lactobacillaceae* and *Bifidobacteriaceae*, and an increase in *E. coli* (Mevissen-Verhage et al., 1985; Balmer et al., 1989; Balmer and Wharton, 1991; Benoni et al., 1993; Tompkins et al., 2001; Lee et al., 2008; Zimmermann et al., 2010; Werner et al., 2011; Dostal et al., 2012a,b, 2014b; Krebs et al., 2013; Jaeggi et al., 2014; Kortman et al., 2014). The large variation in the effects of iron on the gut microbiota composition in humans and animals may be explained by differences in experimental models, and host factors such as iron status, intestinal immune function, dietary habits and the environmental setting. Importantly, *in vitro* models of the colon allow the reproducible testing of the effects of iron on the microbiota in isolation, by eliminating any influence of the host. On the other hand, the most important limitation is the absence host feedback mechanisms that normally influence microbiota composition and activity (Sekirov et al., 2010; Hooper et al., 2012). Also inoculum preparation from feces influences the microbiota. It should be noted that colon models can never establish intestinal conditions identical to those in the host. Therefore, inoculum preparation and inoculation in the model will always result in a newly balanced microbiota. Although these *in vitro* models have their limitations, they have also been shown to closely mimic the *in vivo* situation in terms of microbial composition and metabolism (Payne et al., 2012; Venema and van den Abbeele, 2013).

Besides the intestinal microbiota composition, the bacterial metabolic activity is also important for gut health. For example, indigestible food components can be metabolized into the main Short Chain Fatty Acids (SCFAs) acetate, propionate and butyrate. These, and especially butyrate, are beneficial for gut health, for example by providing energy to colonocytes and by exerting anti-inflammatory effects (Macfarlane and Macfarlane, 2011). In contrast, protein fermentation can result in toxic or potentially toxic metabolites such as ammonia, Branched Chain Fatty Acids (BCFAs) (e.g., isobutyrate and isovalerate), and phenolic compounds (Macfarlane and Macfarlane, 2012; Nyangale et al., 2012). Although the mechanisms of action remain unknown so far, it has recently been shown that iron can influence bacterial metabolic activity (Dostal et al., 2012a,b, 2014b). The *in vivo* levels of butyrate and propionate were lower during luminal iron deficiency in rats, which could be restored by iron repletion (Dostal et al., 2012a). *In vitro*, butyrate and propionate production were most clearly impaired during very low iron conditions, while conversely, the production of these SCFAs was not stimulated by high iron conditions. The production of BCFAs as a result of protein fermentation was decreased under low iron conditions, but in contrast to the production of SCFAs, this proteolytic activity did increase under high iron conditions (Dostal et al., 2012b). Interestingly, a recent study in rats showed that supplementary iron increased cecal SCFA levels not only when compared to an iron-deficient diet, but also when compared to a control diet (Dostal et al., 2014b). However, the latter rat study did not specifically report on the production of BCFAs. Together, these studies suggest that iron supplementation may have health-promoting effects via an increase in microbial SCFA production, but potentially also deleterious effects by increasing toxic protein fermentation. Thus, the net effects of iron preparations on gut microbial metabolism remain inconclusive and need further exploration with both a targeted and metabolomic approach. In the present study we explore the effect of multiple iron sources and doses; i.e., 50 or 250 μmol/L ferrous sulfate, 50 or 250 μmol/L ferric citrate, or 50 μmol/L hemin, on the composition and metabolic activity of a microbiota that is representative for the *in vivo* human gut microbiota. To mimic the human *in vivo* conditions, we use the well-suited and highly controlled TNO's Intestinal Model 2 (TIM-2) for the human large intestine (Minekus et al., 1999; Maathuis et al., 2009).

## Results

### The influence of iron on the microbiota composition

The microbiota composition was analyzed by 16S rDNA sequencing, microarray (I-Chip), and metagenomic sequencing. The main results are shown below, and are complemented by information on total bacteria and microbiota diversity in Supplementary Data 2. With a microbial density of approximately 4 × 10^9^ cells/g in the TIM-2 model, it closely reflects the densities found in the proximal colon (10^10^–10^11^), whereas microbial densities in feces are generally about two orders of magnitude higher (Payne et al., 2012).

#### Microbiome analysis by 16S rDNA sequencing

**Figure 1:**
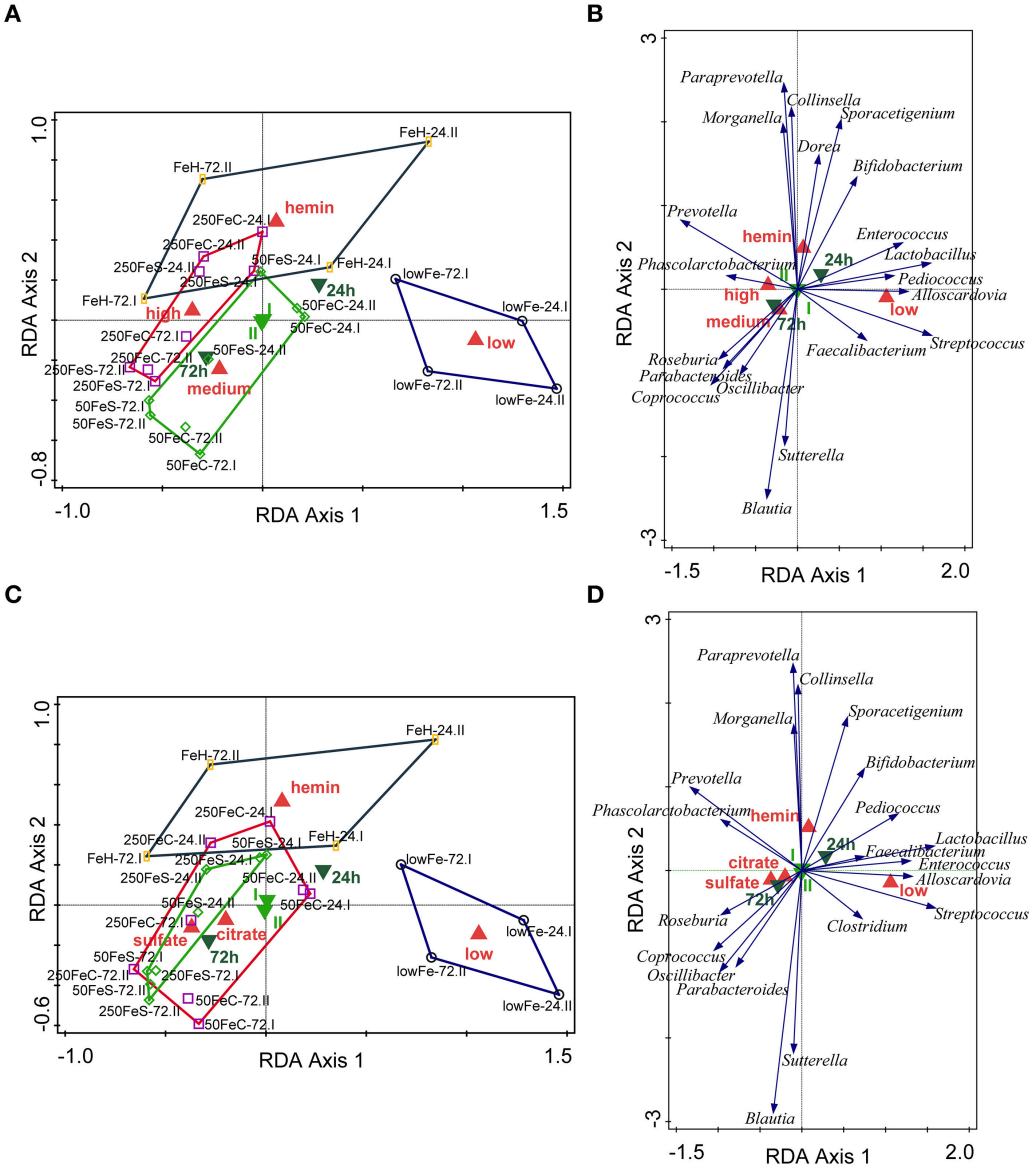
**Multivariate redundancy analysis (RDA) of the microbiota composition**. The conditions were grouped at iron level: low (endogenous Fe; *n* = 4), medium (pooled 50FeS and 50FeC conditions; *n* = 8), high (pooled 250FeS and 250FeC conditions; *n* = 8) and hemin (50 μmol/L hemin; *n* = 4). RDA was performed using Canoco 5.0. Taxonomic composition at the genus level was used as response data, iron level as explanatory variable, time point and experiment as supplementary variables. The variation explained by the ordination axis is significantly higher than random (*p* = 0.04; permutation test). Total variation explained was 33.3% **(A,B)**. To study differences between iron sources, conditions were grouped at iron source: low (*n* = 4), sulfate (pooled 50FeS and 250FeS; *n* = 8), citrate (pooled 50FeC and 250FeC; *n* = 8), and hemin (*n* = 4). Taxonomic composition at the genus level was used as response data, iron source as explanatory variable, time point, and experiment as supplementary variables. The variation explained by the ordination axis is significantly higher than random (*p* = 0.032; permutation test). Total variation explained was 32.5% **(C,D)**. Red symbols represent iron level or iron source classes, light green samples the two replicate experiments and dark green symbols the two time points. Open symbols are the individual samples. The colored lines are envelopes connecting samples of the same iron level or iron source. Length of arrows reflects significance. Classified sample diagrams in **(A,C)**, Taxa—metadata biplots in **(B,D)**.

**Figure 2:**
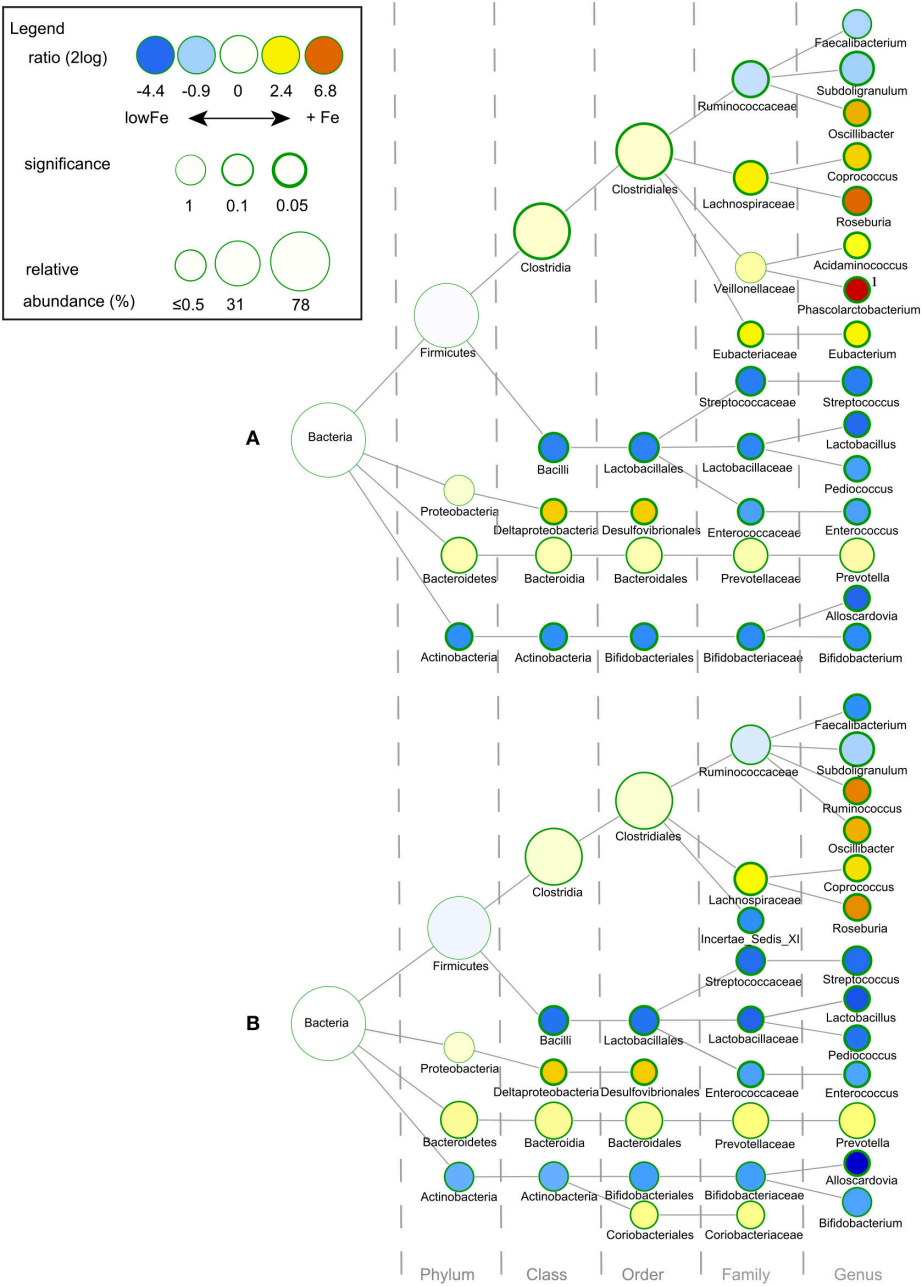
**Differences in gut microbial composition between the lowFe control and the medium and high Fe and conditions at 72 h**. Nodes represent taxa; edges link the different taxonomic levels. The fold difference is calculated as the 2log of the ratio of the relative abundance in the lowFe (*n* = 2) and mediumFe (*n* = 4) conditions **(A)** or in the lowFe and highFe (*n* = 4) conditions **(B)** (0, no difference between groups; 1, twice as abundant in mediumFe/highFe, etc.). In this explorative analysis, the significance is expressed as the *p*-value of a Mann–Whitney *U*-test. The node-size corresponds to the relative abundance. Taxa (that is, nodes) were included in this visualization when the fold difference met a significance level of *p* < 0.1, or when the taxon had a child (that is, more specific taxonomic classification) meeting this criterion. ^1^ *Phascolarctobacterium* was not detected within the lowFe condition and has therefore an estimated 2log fold difference of 10.

We determined the microbiome composition by analyzing on average 3445 bacterial 16S rDNA sequences per sample, obtained by pyrosequencing. Multivariate redundancy analysis (RDA) showed that the different iron levels resulted in a distinct non-overlapping microbiota composition (*p* = 0.040; Figure 1A), while iron supplementation as ferrous sulfate (FeS) or ferric citrate (FeC) resulted in a similar microbiota profile, but that was distinct from that of the endogenous iron (lowFe) and hemin (FeH) conditions (*p* = 0.032; Figure 1C). The main differences between the microbiome of the lowFe controls and the conditions with mediumFe and highFe at 72 h are summarized in Figures 2A,B, respectively. These analyses revealed that the genera *Lactobacillus, Bifidobacterium*, and *Streptococcus* were less abundant in the mediumFe and highFe conditions compared to the lowFe controls and that *Roseburia* and *Coprococcus*, and to a lesser extent, *Prevotella* were more abundant in the mediumFe and highFe conditions. This is consistent with the outcome of the RDA, where these same taxa separated the lowFe from the mediumFe and highFe conditions (Figures 1B,D). Differences between the mediumFe and highFe conditions and differences between the lowFe and FeH conditions are shown in Supplementary Figure 5.

#### Microbiome analysis by I-Chip and metagenomic sequencing

To support and validate the data from 16S rDNA sequencing, we investigated the changes of taxa over time and the effects of iron on certain species by using an I-Chip microarray platform for the analysis of the microbial community in the TIM-2 lumen at every time point (0, 24, 48, and 72h). Moreover, shotgun metagenomic sequencing allowed taxonomic analysis of the duplicate samples of lowFe and 50FeS at 72 h, this was also used to support the 16S rDNA sequencing data.

In both the I-Chip and metagenomic datasets, *Prevotella* was markedly higher in all conditions with supplementary iron at 72 h. I-Chip data furthermore showed that *Prevotella* generally increased in the conditions with iron with a small decrease after 48 h, while its levels remained relatively low and stable in the lowFe condition. Metagenomic analysis also showed that *Prevotella* levels were higher in 50FeS compared to lowFe at 72 h (2log ratio: 7.07; *p* = 0.006; Supplementary Figure 6). Next, I-Chip analysis showed a gradual increase of *Roseburia* in the 50FeS, 250FeS, and 50FeC conditions and metagenomic analysis confirmed that specifically *Roseburia intestinalis* was more abundant at 72 h in 50FeS compared to lowFe (2log ratio: 2.01; *p* = 0.073; Supplementary Tables 1, 2). 16S rDNA sequencing showed that the sulfate-reducing bacteria (SRB) member *Desulfovibrionales* (order) was more abundant in the conditions with iron at 72 h (Figures 2A,B). This is consistent with the metagenomic analysis, where the genus *Desulfovibrio* (2log ratio: 3.08; *p* = 0.042) and specifically the species *D. desulfuricans* (2log ratio: 3.66; *p* = 0.043) and *D. piger* (2log ratio: 1.33; *p* = 0.030) were more abundant in the 50FeS condition compared to lowFe (Supplementary Table 2). Interestingly, although genus *Bacteroides* as a group was not significantly more abundant in the iron groups, several *Bacteroides* spp. were significantly more abundant in 50FeS compared to lowFe, including the opportunistic pathogen *Bacteroides fragilis* (2log ratio: 1.79; *p* = 0.029), which was also apparent from the I-Chip analysis, mainly for the FeS conditions (Supplementary Tables 1, 2).

The metagenomic shotgun sequencing also detected sequences from outside the bacterial domain, including Archaea, Eukaryota, and a small minority of viral sequences. Archaea as a group were relatively more abundant in the 50FeS group than in the lowFe group at 72 h (2log ratio: 4.90; *p* = 0.095). Specific Archaea members that were more abundant included *Methanosphaera* (2log ratio: 6.49; *p* = 0.001)*, Methanobrevibacter* (2log ratio: 7.27; *p* = 0.091) and *Methanobacteria* (2log ratio: 7.24; *p* = 0.093; Supplementary Table 2).

### The influence of iron on the microbial metabolome

Iron is involved in many metabolic processes and is indeed known to influence gut bacterial metabolism, such as the production of SCFAs. Iron-induced changes in the gut microbial composition may also affect gut microbial metabolic activity. Therefore, it is important to explore the effects of supplementary iron on the gut microbial metabolome.

#### Short chain fatty acid and branched chain fatty acid levels

To study the effect of iron on the production of the main SCFA and BCFA, the cumulative amounts of these metabolites were determined in the lumen and dialysate. Analysis showed that there was no significant effect of iron supplementation on acetate production. However, propionate production was doubled in highFe compared to lowFe (*p* < 0.05) and propionate levels were also significantly higher in highFe compared to mediumFe (*p* < 0.05). Butyrate production was similar in all conditions, but was lowest in FeH, which was different from mediumFe (*p* < 0.05; Table 1). Iron source had no significant impact on SCFA levels, except for a lower butyrate level in FeH compared to FeC (Table 1). Although the absolute valerate content was low compared to the other SCFAs, it showed a significant increase under 250FeS compared to lowFe (*p* < 0.05). The valerate level in 250FeS was also significantly higher than in FeH and 50FeC (*p* < 0.05 for both; Table 1). BCFA production was markedly increased in FeS compared to lowFe and FeH, and also compared to FeC (all *p* < 0.05), which implies that ferrous sulfate stronger stimulated protein fermentation compared to ferric citrate. Interestingly, iron level (medium vs high) had no significant impact on BCFA levels (Table 1).

**Table 1:** **Effect of iron on the microbial production of SCFAs, and metabolites associated with proteolytic and saccharolytic activity**.

		**SCFA (saccharolytic mainly)**			**Proteolytic activity associated**				**Saccharolytic activity associated**			
		**Acetate (mmol)**	**Butyrate (mmol)**	**Propionate (mmol)**	**Valerate (mmol)**	**Isovalerate (mmol)**	**Isobutyrate (mmol)**	**Ammonia (mmol)**	**Lactate (a.u.)**	**Succinate (a.u.)**	**Formate (mmol/L)**	**Ethanol (mmol/L)**
Single conditions	lowFe	66.8 (66.0-67.6)	40.4 (37.2-43.6)	14.1 (11.5-16.7)	0.0 a (0.0-0.0)^*^	0.1 (0.0-0.3)^*^	0.0 (0.0-0.0)^*^	30.5 (29.1-31.9)	100.0 a (66.4-133.6)	100.0 a (8.6-191.4)	2.1 a (2.1-2.1)	2.1 a (2.0-2.2)
	50FeS	84.2 (73.6–94.4)	46.1 (43.1-49.0)	17.7 (14.8-20.5)	0.9 ab (0.7-1.1)	1.5 (1.3-1.6)	0.6 (0.6-0.7)	52.1 (48.7-55.5)	7.3 b (7.2-7.4)	202.3 ac (49.4-355.3)	0.4 b (0.4-0.5)	0.4 b (0.3–0.6)
	250FeS	83.8 (79.7-87.9)	46.0 (43.9-48.1)	26.1 (21.1-31.2)	1.5 b (1.2-1.8)	1.7 (1.5-2.0)	0.7 (0.6-0.7)	57.5 (56.2-58.8)	6.1 b (5.5-6.6)	4.7 a (2.3–7.1)	0.4 b (0.4-0.4)	0.2 b (0.1-0.2)
	50FeC	76.2 (71.6-80.8)	53.0 (51.2-54.8)	16.0 (15.9-16.1)	0.2 a (0.1-0.3)	0.6 (0.5-0.7)	0.1 (0.1-0.1)	50.3 (48.0-52.7)	5.9 b (4.4-7.4)	632.8 b (627.4-638.1)	0.4 b (0.4-0.5)	1.6 a (1.3-1.8)
	250FeC	83.0 (74.9-91.1)	46.4 (43.8-49.0)	30.3 (28.4-32.3)	0.9 ab (0.5-1.3)	0.7 (0.1-1.3)	0.3 (0.0-0.5)^*^	47.7 (36.6-58.7)	6.3 b (6.3-6.3)	13.3 a (6.3-20.3)		
	FeH	80.6 (76.6-84.6)	37.4 (34.5-40.3)	28.0 (23.1-33.0)	0.1 a (0.1-0.2)	0.3 (0.1-0.5)	0.1 (0.0-0.1)^*^	40.7 (35.7-45.7)	7.0 b (6.2-7.7)	527.9 bc (524.2-531.6)	0.5 b (0.5-0.5)	1.6 a (1.4-1.8)
Iron level (grouped)	lowFe	66.8 (66.0-67.6)	40.4 ab (37.2-43.6)	14.1 a (11.5-16.7)	0.0 (0.0-0.0)^*^	0.1 (0.0-0.3)^*^	0.0 (0.0-0.0)^*^	30.5 a (29.1-31.9)	100.0 a (66.4-133.6)	100.0 ab (8.6-191.4)		
	MediumFe	80.2 (71.6-94.9)	49.5 a (43.1-54.8)	16.8 ab (14.8-20.5)	0.6 (0.1-1.1)	1.0 (0.5-1.6)	0.4 (0.1-0.7)	51.2 b (48.0-55.5)	6.6 b (4.4-7.4)	417.5 a (49.4-638.1)		
	HighFe	83.4 (74.9-91.1)	46.2 ab (43.8-49.0)	28.2 c (21.1-32.3)	1.2 (0.5-1.8)	1.2 (0.1-2.0)	0.5 (0.0-0.7)^*^	52.6 b (36.6-58.8)	6.2 b (5.5-6.6)	9.0 b (2.3-20.3)		
	FeH	80.6 (76.6-84.6)	37.4 b (34.5-40.3)	28.0 abc (23.1-33.0)	0.1 (0.1-0.2)	0.3 (0.1-0.5)	0.1 (0.0-0.1)^*^	40.7 ab (35.7-45.7)	7.0 b (6.2-7.7)	527.9 a (524.2-531.6)		
Iron source (grouped)	LowFe	66.8 (66.0-67.6)	40.4 ab (37.2-43.6)	14.1 (11.5-16.7)	0.0 a (0.0-0.0)^*^	0.1 a (0.0-0.3)^*^	0.0 a (0.0-0.0)^*^	30.5 a (29.1-31.9)	100.0 a (66.4-133.6)	100.0 (8.6-191.4)		
	FeS	84.0 (73.6-94.4)	46.0 ab (43.1-49.0)	21.9 (14.8-31.2)	1.2 b (0.7-1.8)	1.6 b (1.3-2.0)	0.6 b (0.6-0.7)	54.8 b (48.7-58.8)	6.7 b (5.5-7.4)	103.5 (2.3-355.3)		
	FeC	79.6 (71.6-91.1)	49.7 a (43.8-54.8)	23.2 (15.9-32.3)	0.5 ab (0.1-1.3)	0.6 a (0.1-1.3)	0.2 a (0.0-0.6)^*^	49.0 ab (36.6-58.7)	6.1 b (4.4-7.4)	323.0 (6.3-638.1)		
	FeH	80.6 (76.6-84.6)	37.4 b (34.5-40.3)	28.0 (23.1-33.0)	0.1 ab (0.1-0.2)	0.3 a (0.1-0.5)	0.1 a (0.0-0.1)^*^	40.7 ab (35.7-45.7)	7.0 b (6.2-7.7)	527.9 (524.2-531.6)		

Cumulative levels in the lumen and dialysate at 72 h (mean+range) of the main SCFAs acetate, butyrate and propionate are given for the single conditions, the grouped mediumFe and highFe conditions, and the grouped FeS and FeC conditions. Cumulative levels at 72 h (mean+range) of valerate, the BCFAs isobutyrate and isovalerate, and ammonia (proteolytic activity associated metabolites) are displayed the same way. The saccharolytic activity associated metabolites lactate, succinate, formate and ethanol were measured by organic acid analysis and/or ^1^H-NMR spectroscopy in the dialysate at 72 h. Lactate and succinate levels (mean+range) are shown relative to the lowFe control which was set at 100%; the average concentrations of lactate and formate in the lowFe condition were 3.3 mmol/L and 0.29 mmol/L respectively, as quantified by ^1^H-NMR spectroscopy. Formate and ethanol levels (mean+range) as determined by ^1^H-NMR spectroscopy are not shown for grouped conditions because the 250FeC condition was not analyzed by ^1^H-NMR spectroscopy. Means of the same metabolite, and within its horizontal category, without a common letter differ significantly (p < 0.05; Tukey's multiple comparison post-hoc test). N = 2 for the single conditions, and n = 4 for the grouped conditions mediumFe, highFe, FeS, and FeC.

* Value of 0 was assigned when a metabolite was below the detection limit.

#### Ammonia levels

Ammonia, a toxic metabolite that originates from protein breakdown, was determined in the lumen and dialysate of TIM-2. Similar to BCFAs, the cumulative ammonia level was increased in all iron conditions and most prominently in the ferrous sulfate conditions. When iron levels were pooled, the ammonia level was higher in both mediumFe and highFe than in lowFe (*p* < 0.05; Table 1). Interestingly, when the iron sources were pooled, only the ferrous sulfate pool showed a significantly higher ammonia level than lowFe (*p* < 0.05), but the ferrous sulfate pool was not significantly different from the ferric citrate pool (Table 1).

#### ^1^H-NMR-spectroscopy and organic acid analysis

^1^H-NMR spectroscopy and GC-MS organic acid analysis were used to explore the microbial metabolome in the dialysate. ^1^H-NMR spectroscopy detected a total of 26 metabolites, of which 20 could be identified and quantified (Supplementary Table 3). Moreover, nine organic acid metabolites could be identified and relatively quantified by GC-MS organic acid analysis. A main effect of iron supplementation was the sharp decrease of lactate compared to the lowFe control (*p* < 0.05), which was detected with both GC-MS and ^1^H-NMR techniques (Table 1). Another abundant metabolite that mainly derives from carbohydrate fermentation, was succinate. Compared to lowFe, succinate content was higher in FeH and 50FeC (*p* < 0.05 for both). Remarkably, the succinate level was higher in 50FeC compared to 50FeS (*p* < 0.05), but was almost absent in both high-iron conditions, with a large difference between 50FeC and 250FeC (*p* < 0.01; Table 1).

**Figure 3:**
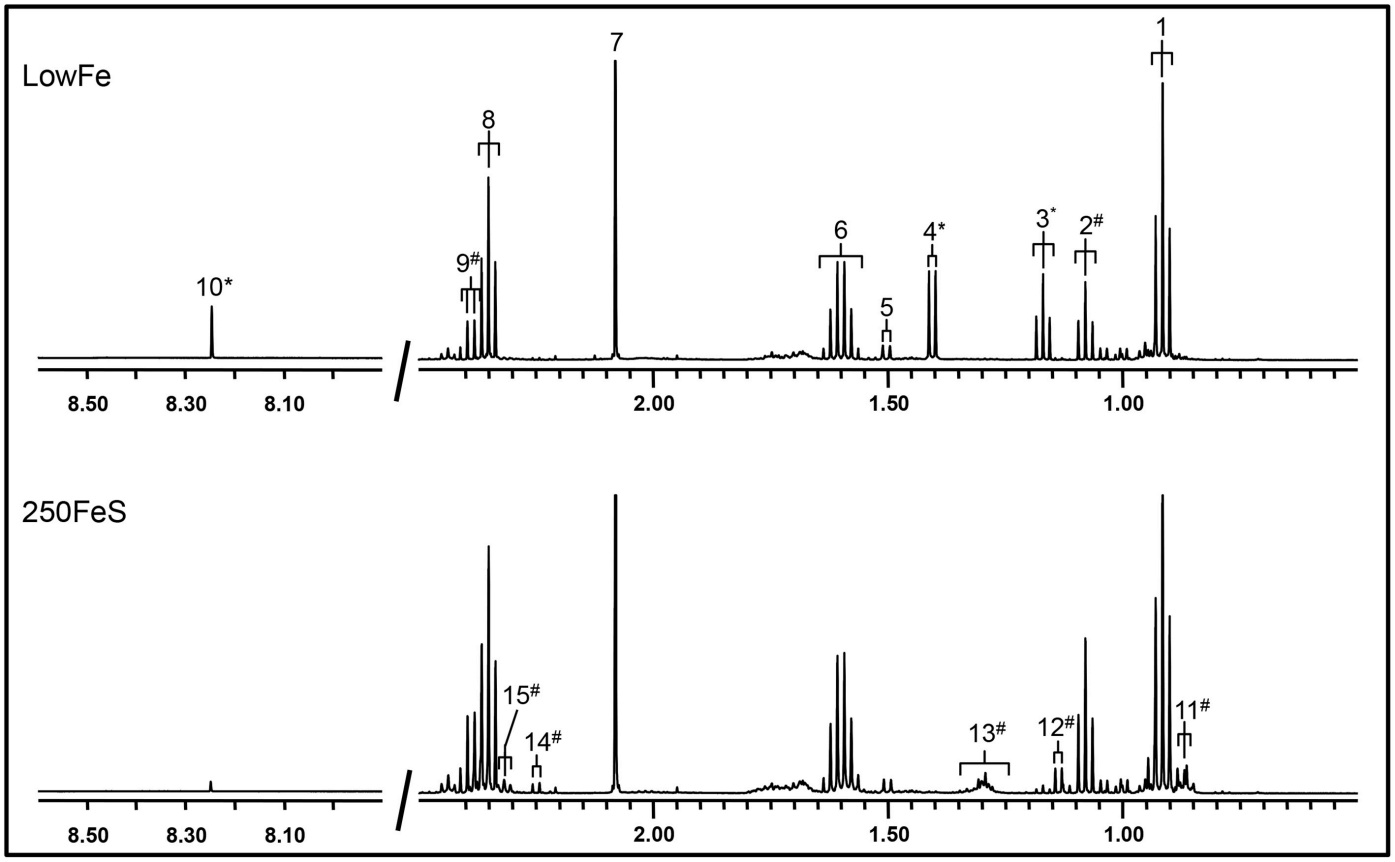
**500 MHz ^1^H-NMR spectra (regions 2.7–0.5 ppm and 8.5–8.0 ppm) of dialysate samples**. ^1^H-NMR spectroscopy at pH 2.50 of the lowFe condition (**upper panel**) and 250FeS condition (**lower panel**) at 72h showed that the SCFAs butyrate and acetate were similar in both conditions, while propionate content was slightly higher in 250FeS. Lactate and ethanol disappeared in the iron condition, while valerate content showed the opposite effect (see also Table 1). Peak assignments: butyrate (1, 6, and 8), propionate (2 and 9), ethanol (3), lactate (4), alanine (5), acetate (7), formate (10), valerate (11, 13, and 15) isobutyrate (12) and isovalerate (14). ^#^increased in the condition with iron; ^*^decreased in the condition with iron. Of both conditions one replicate is shown, which were representative for their duplicate. Also, the results of the ^1^H-NMR method correlate well with the results of both the GC-MS organic acid analysis and the SCFA/BCFA GC-method.

Part of a ^1^H-NMR spectrum is depicted in Figure 3 where lowFe and 250FeS are compared. ^1^H-NMR spectroscopy thus confirms the increase in valerate as mentioned above. This technique also revealed an almost equal decrease of approximately four-fold in formate content for all iron-supplemented conditions (250FeC not assessed) compared to lowFe (*p* < 0.001; Table 1). Further, metabolite profiling showed a decrease in ethanol content in both ferrous sulfate conditions compared to lowFe (both *p* < 0.01) and also when compared to FeH and 50FeC (both *p* < 0.05; Table 1).

**Figure 4:**
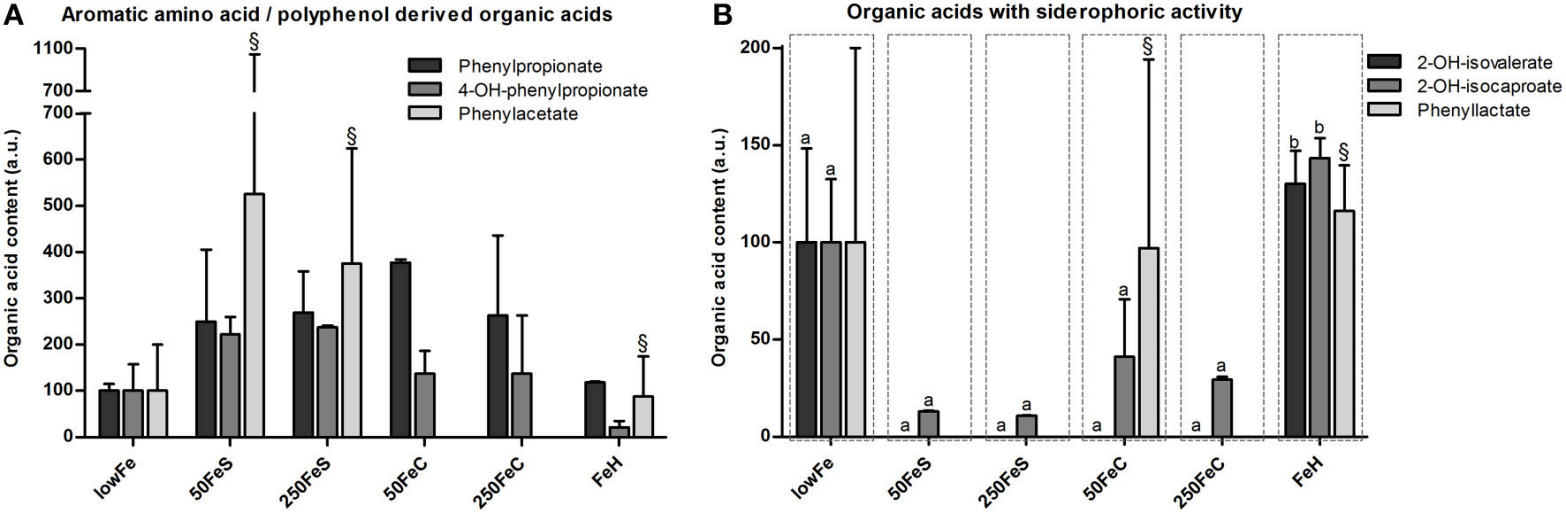
**Effect of iron on organic acid levels associated with proteolytic or polyphenol metabolism and on organic acids with siderophoric activity**. Concentrations (mean+range) of phenylpropionate, 4-hydroxy-phenylpropionate and phenylacetate at 72 h are displayed relative to the lowFe condition which was set at 100%.^§^We note that phenylacetate levels included traces of glycerol in one biological replicate of 50FeS, 250FeS, and FeH, but which were negligible amounts relative to the phenylacetate levels **(A)**. Concentrations (mean+range) of metabolites with siderophoric activity; 2-hydroxy-isovalerate, 2-hydroxy-isocaproate, and phenyllactate at 72 h are displayed relative to the lowFe condition which was set at 100%.^§^We note that phenyllactate levels included negligible traces of a unknown metabolite in 1 biological replicate of 50FeC and FeH. Means (*n* = 2; compared by Tukey's multiple comparison *post-hoc* test) of the same metabolite without a common letter differ significantly (*p* < 0.05) **(B)**.

Among the organic acids identified, most were aromatic amino acid or polyphenol-derived and they generally showed an increase upon iron supplementation. Phenylpropionate content tended to be higher in both ferrous sulfate conditions, and also in the ferric citrate conditions (not significant; Figure 4A). Phenylacetate levels appeared to be higher 50FeS and 250FeS compared to lowFe, but it was not detected in 50FeC and 250FeC (Figure 4A). 4-Hydroxy-phenylpropionate content was mainly increased in both ferrous sulfate conditions (not significant; Figure 4A). Although only in small amounts, 3-hydroxy-phenylpropionate was exclusively detected in 250FeS and was considered to be absent in all other conditions (data not shown). Although none of these effects were statistically significant, they support our findings that proteolytic activity is stimulated by iron supplementation.

#### Siderophoric metabolites detected by organic acid analysis

Other amino acid-derived metabolites were categorized as compounds with siderophore activity. 2-Hydroxy-isovalerate was only detected in substantial amounts in the dialysate of lowFe and FeH (Figure 4B). 2-Hydroxy-isocaproate content showed a similar pattern and was significantly lower in the pooled ferrous sulfate, the pooled ferric citrate, the pooled mediumFe and the pooled highFe conditions compared to lowFe and FeH (all *p* < 0.05). Phenyllactate content was relatively variable within the biological duplicates, nevertheless it was exclusively below the detection limit in all ferrous sulfate replicates and also in 250FeC (Figure 4B). These data support a potential siderophore function of these metabolites in a low-iron environment.

### Correlations of metabolite levels with the microbiome

It is conceivable that the changes in microbial metabolism can result from an altered microbiome composition. However, microbial metabolism may, in theory, also respond to iron without a change in abundances of certain taxa. As a first attempt to link changes in the microbiome composition to the marked changes in microbial metabolism we aimed to correlate SCFA, butyrate, propionate, acetate, BCFA and ammonia levels with the relative abundance of bacterial taxa of the microbiome as analyzed by 16S rDNA pyrosequencing.

#### Short chain fatty acids

**Figure 5:**
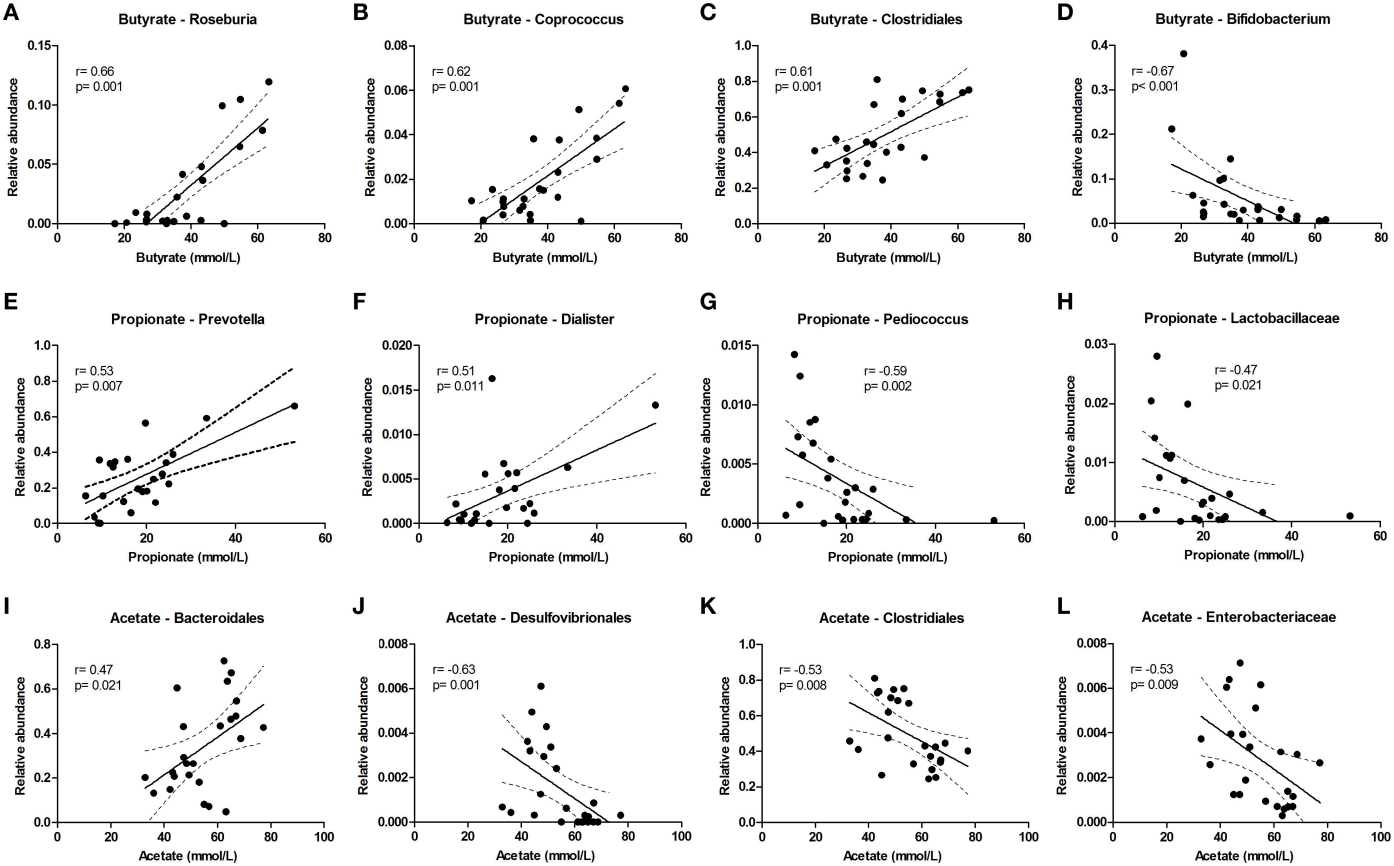
**Bacterial taxa that correlate with luminal acetate, butyrate, and propionate levels**. The relative abundance (fraction of the total 16S rDNA; e.g., 0.01 = 1%) of bacterial taxa in all samples at 24 and 72 h (*n* = 24) (as determined by 16S rDNA pyrosequencing) was correlated with luminal butyrate **(A–D)**, propionate **(E–H)**, and acetate **(I–L)** levels in the same samples. Best fit lines with 95% confidence bands were generated by linear regression analysis. Spearman *r*- and *p*-value are displayed for each graph. This figure shows the taxa that correlated best, additional taxa are shown in Supplementary Figure 7.

Butyrate levels were positively correlated with *Roseburia* and *Coprococcus*, which both are known for the production of butyrate, and with Clostridiales, while *Bifidobacterium* correlated negatively (Figures 5A–D). Propionate correlated with *Prevotella* and *Dialister*, and was inversely correlated with *Pediococcus* and *Lactobacillaceae* (Figures 5E–H). Acetate levels could be correlated to *Bacteroidales* and were inversely correlated to *Desulfovibrionales, Clostridiales* and *Enterobacteriaceae* (Figures 5I–L). The sum of luminal butyrate, propionate and acetate levels in the lumen at 24 and at 72 h correlated positively with *Roseburia*, and was inversely correlated with *Bifidobacteriaceae* (Supplementary Figure 7). Spearman r and *p*-value of a Spearman rank test are depicted within the figures.

#### Branched chain fatty acids and ammonia

**Figure 6:**
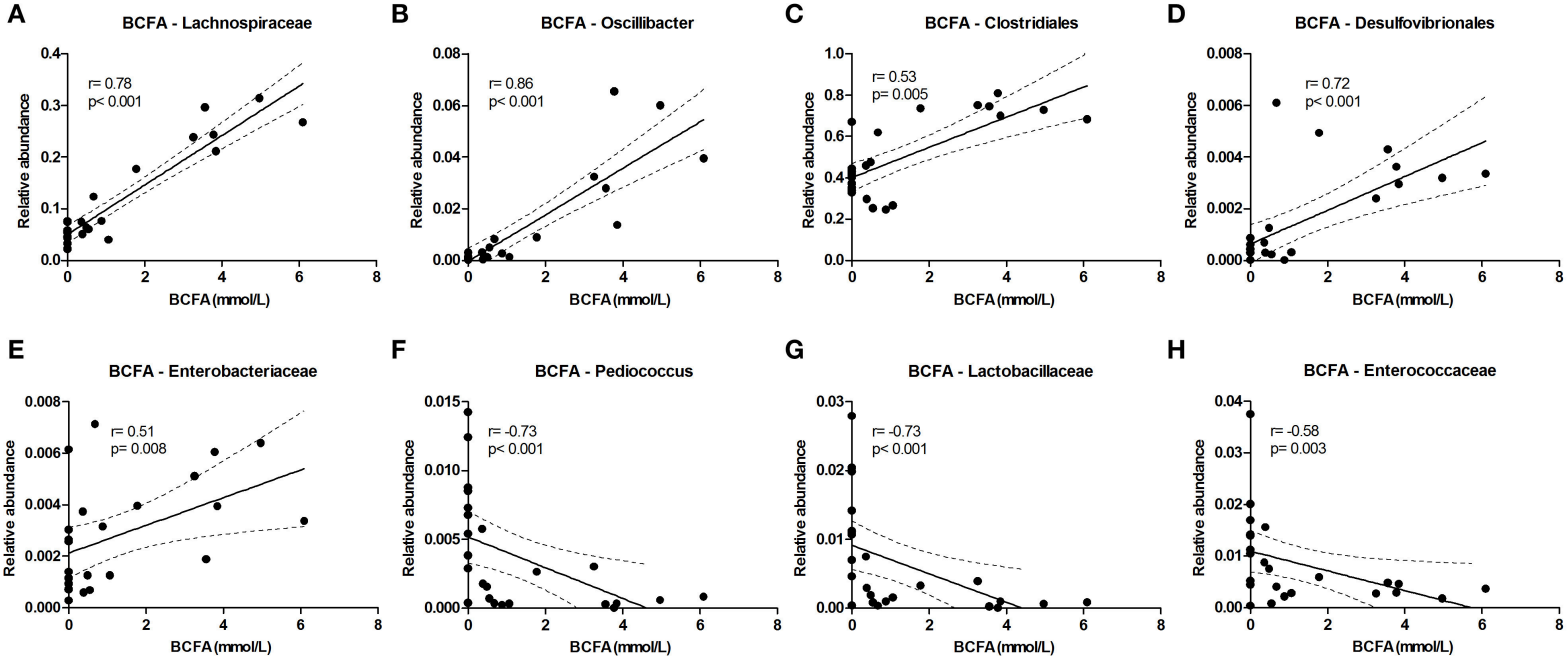
**Bacterial taxa that correlate with BCFA levels**. The relative abundance (fraction of the total 16S rDNA; e.g., 0.01 = 1%) of bacterial taxa in all samples at 24 and 72 h (*n* = 24; as determined by 16S rDNA pyrosequencing) was correlated with the sum of isobutyrate and isovalerate (collectively called BCFAs) levels in the same samples. **(A–E)** Show positive correlations while **(F–H)** show inverse correlations. Best fit lines with 95% confidence bands were generated by linear regression analysis. Spearman *r*-and *p*-value are displayed for each graph. This figure shows the taxa that correlated best, additional taxa are shown in Supplementary Figure 8.

The level of the proteolytic metabolites ammonia and BCFA could be correlated to the following taxa. *Lachnospiraceae* (among which were *Roseburia* and *Coprococcus*) were strongly correlated with BCFA levels, and also *Oscillibacter, Clostridiales, Desulfovibrionales*, and *Enterobacteriaceae* correlated positively with BCFA levels. Conversely, *Pediococcus, Lactobacillaceae*, and *Enterococcaceae* were inversely correlated (Figure 6). Similar results were found for cumulative ammonia levels as they strongly correlated with *Lachnospiraceae* (among which were *Roseburia* and *Coprococcus*), and also with *Clostridiales, Desulfovibrionales, Oscillibacter, Enterobacteriaceae*, and *Dialister*. Furthermore, cumulative ammonia levels were inversely correlated with *Pediococcus, Bacteroides* and *Lactobacillaceae* (Supplementary Figures 8A–K).

### Metagenomic analysis: mapping the metabolome to the metagenome

To deduce where the changes in microbial metabolism may originate from, we correlated the relative abundance of bacterial taxa to metabolite levels, and moreover, we linked the metabolite levels to metabolic pathways identified in the metagenome. Furthermore, metagenomic analysis allowed us to explore what other functions and hallmarks were over- or underrepresented in the lowFe and 50FeS microbial communities.

#### General metagenomic annotations

To investigate the effect of iron on the functional potential of the gut microbial community, we analyzed a total of 69.9 and 59.8 million quality trimmed metagenomic sequencing read pairs (average single end read length: 207 base pairs) for the lowFe and 50FeS replicates at 72 h, respectively. A total of 43.2 million sequences (32.5%) were aligned to the human gut microbial gene catalog (Qin et al., 2010) allowing them to be functionally characterized in terms of the SEED subsystems and KEGG orthology annotations An overview of the functional composition (SEED subsystems) of the lowFe and 50FeS conditions is shown in Supplementary Figure 9.

The functions that were most strongly affected by the iron treatment were related to the “Motility and Chemotaxis” level-1 subsystem, that was overrepresented in 50FeS compared to lowFe (2log ratio: 0.40; *p* = 0.044). The more detailed annotations provided by the SEED level-2 subsystems showed that this could be attributed to the categories “Flagellar Motility” (2log ratio: 0.80; *p* = 0.053), “Flagellum” (2log ratio: 0.67; *p* = 0.054) and “Bacterial Chemotaxis” (2log ratio: 0.19; *p* = 0.091; Supplementary Table 4). Moreover, this was also supported by the many flagellar and chemotaxis related KEGG orthologs that were significantly enriched in 50FeS (Supplementary Table 5). One subsystem of special interest in the context of this study was “Iron Acquisition and Metabolism.” Although there was no net difference found of the level-1 subsystem, we found that the level-2 subsystem “Transport of Iron” was enriched in the 50FeS condition (2log ratio: 0.34; *p* = 0.001; Supplementary Table 4), as may be expected for the condition with supplementary iron.

#### Saccharolytic and proteolytic related subsystems

With metagenome analysis we aimed to link the marked metabolic changes described above to the enrichment or depletion of SEED subsystems and KEGG orthologs to find out which mechanisms were possibly responsible for the metabolic changes. This is rather complex as many enzymatic systems can work in two directions and because the balance in microbial production and utilization of metabolites is often difficult to predict. This analysis nevertheless provided some clues which might explain part of the metabolic pattern as we observed in the lumen/dialysate. Metabolite analysis pointed at a shift from carbohydrate fermentation toward protein fermentation upon iron supplementation. Subsystem data showed that the level-2 subsystem “Protein Degradation” was enriched in 50FeS (2log ratio: 0.39; *p* = 0.008). This involved enrichment of aminopeptidases (EC 3.4.11.x) (KEGG orthologs; Supplementary Table 5), which may be involved in exogenous protein fermentation. Carbohydrate related subsystems that may support a decreased carbohydrate fermentation include level-2 subsystems “Fructooligosaccharides (FOS) and Raffinose Utilization” (2log ratio: −0.20; *p* = 0.071) and “Beta-glucoside Metabolism” (2log ratio: −0.64; *p* = 0.022), both of which were reduced in 50FeS (Supplementary Table 4). The apparent shift toward a proteolytic profile was characterized by an increase in ammonia, BCFA and aromatic metabolites. We attempted to link these specific changes to the subsystems and KEGG orthologs as specified below.

##### Ammonia related subsystems

Level-1 subsystem “Amino Acids and Derivatives” showed that its level-2 subsystems “Lysine Degradation” (2log ratio: −0.25; *p* = 0.045) was underrepresented in 50FeS, while “Tryptophan Synthesis” and “Cysteine Biosynthesis” were enriched (2log ratio: 0.41; *p* = 0.005 and 0.36; *p* = 0.012, respectively). This would typically lead to decreased amounts of ammonia, but in contrast, “Glutamate Dehydrogenase” (2log ratio: −0.28; *p* = 0.061) and “Glutamine Synthetases” (2log ratio: −0.36; *p* = 0.106), which may assimilate ammonia, were underrepresented in 50FeS. Additionally, subsystem “Ammonia Assimilation” was underrepresented in 50FeS (2log ratio: Ammonia related subsystems 0.24; *p* = 0.037) which could fit with the increase of ammonia in this condition (Supplementary Table 4). Annotation of the metagenomic sequencing reads to KEGG orthologs showed that nitrogen fixation genes *NifA* and *NifB* were enriched in 50Fe, which also fits with increased levels of ammonia (Supplementary Table 5).

##### Branched chain fatty acid-related subsystems

Level-1 subsystem “Amino Acids and Derivatives” showed that its level-2 subsystem “Ketoisovalerate Oxidoreductase” was enriched in 50FeS (2log ratio: 0.45; *p* = 0.050; Supplementary Table 4). This ferredoxin-type enzyme is involved in the degradation of valine, leucine and isoleucine degradation which can result in the production of BCFAs. Annotation of the sequences to KEGG orthologs also showed that ketoisovalerate oxidoreductase (EC 1.2.7.7) was enriched in the 50FeS condition compared to lowFe (Supplementary Table 5).

##### Aromatic compounds related subsystems

Level-1 subsystem “Amino Acids and Derivatives” showed that its level-2 subsystem “Aromatic Amino Acid Interconversions with Aryl Acids” was enriched in 50FeS (2log ratio: 0.76; *p* = 0.040). This ferredoxin-type system converts aromatic amino acids to aryl acids, among which is phenylacetate and possibly also other phenylalanine derivatives like phenyllactate and phenylpropionic acid. Similarly, level-1 subsystem “Metabolism of Aromatic Compounds” showed an enrichment of the level-2 subsystem “Aromatic Amin Catabolism” (2log ratio: 0.55; *p* = 0.088) which may have contributed to the higher levels of phenyl derivatives in the 50FeS condition. In contrast, subsystem “Homogentisate Pathway of Aromatic Compound Degradation” was mildly underrepresented in 50FeS (2log ratio: −0.27; *p* = 0.014; Supplementary Table 4).

### Cytotoxicity of the fecal water

**Figure 7:**
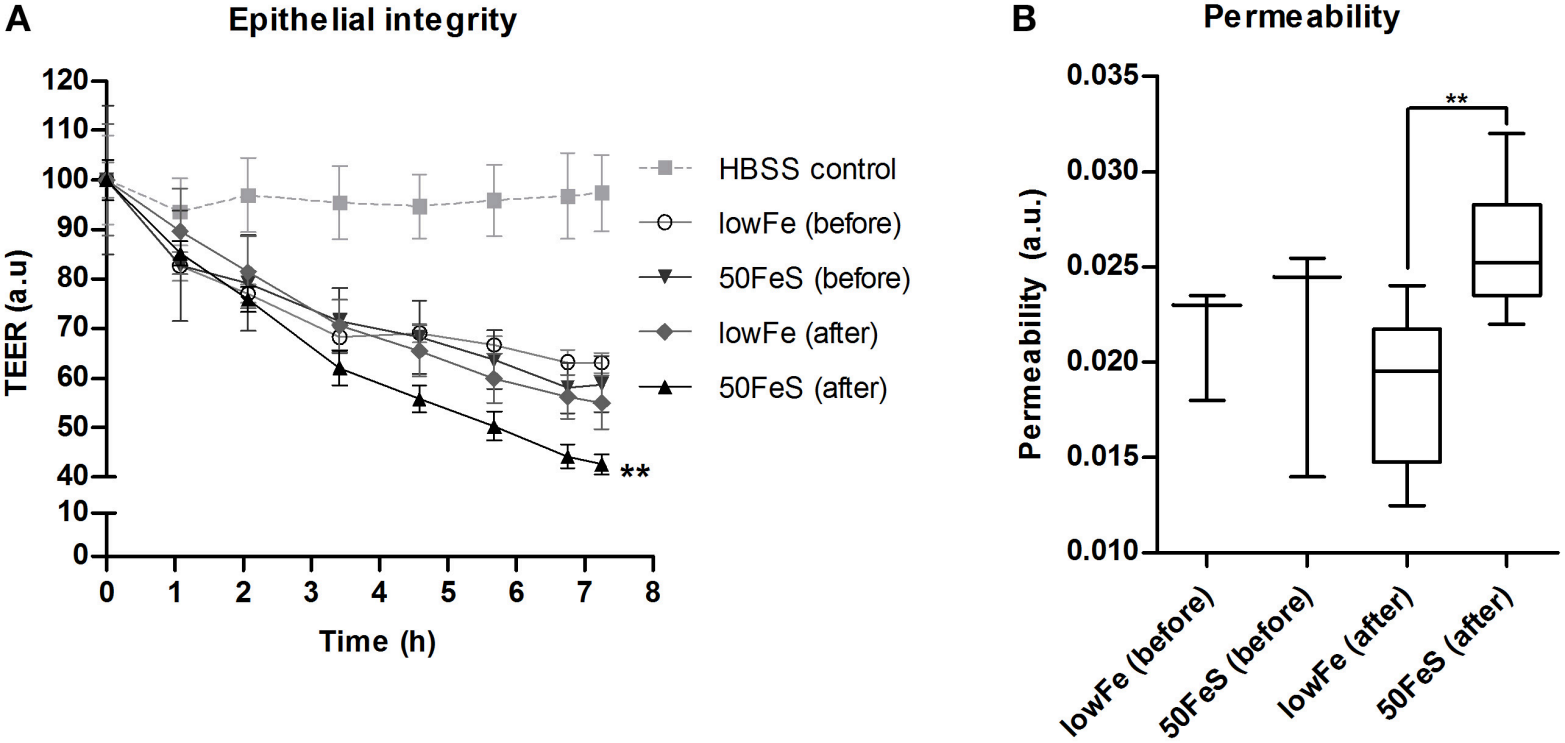
**Toxicity of the microbial metabolome for an intestinal monolayer**. Effect of dialysis liquids (before exchange in TIM-2; not containing microbial metabolites) and dialysates (after exchange in TIM-2; at 72 h) of the lowFe condition and 50FeS condition on the integrity and permeability of a Caco-2 monolayer. Epithelial integrity is displayed as relative transepithelial electrical resistance (TEER) which was regularly recorded during 7 h incubation. HBSS was used as a standard maintaining buffer which should keep the TEER at a constant high level. The decrease in epithelial integrity of the 50FeS dialysate was significantly faster compared to the lowFe dialysate as analyzed by linear regression. We note that iron was removed from the dialysis liquids and dialysates prior to applying them to the epithelial monolayers **(A)**. Epithelial permeability as determined by the flux of phenol red from the apical side to the basolateral side, analyzed by an unpaired *t*-test **(B)**. ^**^p < 0.01 *n* = 3 for the dialysis liquids before exchange in TIM-2 and *n* = 6 for the dialysates after exchange; at 72 h in TIM-2.

Based on the metabolome data, the toxicity of the fecal water was increased with iron, especially in the ferrous sulfate conditions. To confirm this, the cytotoxicity of the 50FeS dialysate toward a tight monolayer of intestinal epithelial Caco-2 cells was assessed. These cytotoxicity tests comprised multiple readouts, i.e., monitoring of the trans epithelial electrical resistance (TEER) (cellular monolayer integrity), phenol red permeability (cellular monolayer permeability), lactate dehydrogenase (LDH) release (cell cytotoxicity), and trypan blue staining (cell death). To exclude the effect of iron itself on the cells, iron was removed from all dialysates prior to the cytotoxicity analyses. Monitoring of TEER showed a deteriorating effect of the dialysis liquids (before exchange in TIM-2) and dialysates (after exchange; sampled at 72 h) in the TIM-2 system when compared to a standard maintaining buffer. Remarkably, the 50FeS dialysate at 72 h showed a larger relative TEER decrease compared to the lowFe dialysate at 72 h (Figure 7A). The slopes as analyzed by linear regression were significantly different (*p* = 0.002). Also note that the lowFe conditions before and after exchange in the TIM-2 system show similar results. Phenol red permeability confirmed these findings as the permeability of the Caco-2 monolayer was shown to be increased after exposure to 50FeS dialysate at 72 h compared to lowFe dialysate at 72 h (*p* = 0.008) (Figure 7B). The decrease in epithelial integrity may be caused by a loss of junction proteins and/or direct toxicity to the cells. Analysis of LDH release and trypan blue staining point at increased cell cytotoxicity and cell death (*p* = 0.081 and *p* = 0.067 respectively; Supplementary Figure 10), but initial loss of junction proteins may also add to the effect.

## Discussion

To find solutions for the unwanted side effects of oral iron administration, such as diarrhea and increased gut inflammation, it is important to investigate the effects of iron on the gut microbiota composition and metabolic activity. Although several studies have reported on the effects of iron on gut microbiota composition, the effects of iron on gut microbial metabolism and its functional potential has remained largely unexplored. We hypothesize that supplementary iron adversely affects gut microbiota composition, and that it may increase microbial protein fermentation and production of SCFAs. It is the first exploration that reports on the effect of multiple iron sources and doses on the microbiome composition, metagenome, and that included profiling of the effects of these iron interventions on the microbial metabolome in both an explorative and targeted manner.

### Preparation of the microbiota and its general characteristics in the TIM-2 model

The present study was done in an *in vitro* model for the human colon. It has been observed that models such as TIM-2 can effectively maintain a stable gut microbiota similar to the donors from whom it was prepared, and that also mimics its functionality (Kovatcheva-Datchary et al., 2009; Rajilic-Stojanovic et al., 2010; Maathuis et al., 2012). Notably, the studies in TIM-2 include a control setup operated in parallel with the test experiments to ensure that the changes observed originate from the treatments itself. A standardized inoculum that is derived from a pool of subjects is generally used in this system, but the use of either a pool of donors or a single donor remains debatable among experts (Kovatcheva-Datchary et al., 2009; Rajilic-Stojanovic et al., 2010; Maathuis et al., 2012). It is questioned whether a pooled inoculum is representative for the abundance and the variety of bacterial species, and their interactions, in the human colon. Consequently, the use of an individual or a mixed inoculum is believed to lead to a degree of variation among experiments. Nevertheless, pooling was done with the purpose of working with a standardized microbiota that can be used for multiple different experiments, and a more diverse population of bacteria that is more representative microbiota for the whole population. In a recent study in TIM-2, we demonstrated that the use of a pool of microbiota for *in vitro* studies does not result in a bacterial community with an aberrant profile and metabolic activity compared to that normally obtained from single donors (Aguirre et al., 2014). Notably, it was previously reported that the effect of arabinoxylan and inulin on an inoculum prepared from pooled fecal samples (in TIM-2) and from an individual volunteer (in SHIME; Simulator of the Human Intestinal Microbial Ecosystem; another *in vitro* colon model) showed a similar metabolic activity of the microbiota in both setups (Van den Abbeele et al., 2013). Therefore, we are convinced that a single pooled inoculum as applied herein (*n* = 1, TIM-2 run in duplicate), is a justified and cost-efficient strategy to profile the effects of a dietary substance on gut microbiota composition and activity in an *in vitro* system.

### Supplementary iron induces a shift toward a less favorable microbiota composition

As expected, iron had a marked effect on microbiome composition. Our data indicate that the high iron doses had an additional effect compared to the medium iron doses. Interestingly, the ferrous sulfate and ferric citrate conditions had a very similar effect on the gut microbiota composition. Comparison of the relative abundance of single bacterial taxa between groups showed that the most apparent differences were the lower relative abundance of *Streptococcus, Lactobacillus*, and *Bifidobacterium* with supplementary iron, which is roughly in line with previous studies for at least the latter two taxa (Kortman et al., 2014). Notably, *Bifidobacterium* and *Lactobacillus* are generally considered beneficial members of the gut microbiota. Interestingly, in our model several other members of the lactic acid bacteria group (to which *Lactobacillus* belongs), i.e., *Streptococcus, Pediococcus*, and *Enterococcus* also showed a decreased relative abundance in response to iron. We did however not observe an increase in *Enterobacteriaceae* or closely related taxa, which was frequently observed in former *in vivo* studies (Mevissen-Verhage et al., 1985; Balmer et al., 1989; Benoni et al., 1993; Lee et al., 2008; Zimmermann et al., 2010; Jaeggi et al., 2014). This could suggest that an increase in *Enterobacteriaceae* depends on host inflammatory responses which they can exploit to their own benefit (Winter et al., 2013). Another explanation that we cannot exclude, is that the specific composition of the here used adult fecal microbiota of Dutch volunteers, which is different from the above mentioned infant or animal microbiota in previous studies, might inhibit overgrowth of this taxon. Instead, we found increased levels of the genera *Roseburia, Coprococcus, Oscillibacter, Prevotella*, and *Desulfovibrio.* We also report that the relative abundance of certain *Bacteroides* species was increased with 50FeS. Among these was *B. fragilis*, an opportunistic and potent pathogen, but that is also important in shaping our immune system (Wexler, 2007). Finally, it appeared from our study that Archaea tend to increase with ferrous sulfate, with *Methanosphaera* being the most significant taxon. Archaea could be involved in the pathogenesis of intestinal diseases, but it was also proposed that their presence is a sign of a healthy gut, because they can thrive on health promoting bacterial metabolites such as SCFAs and formate (Jarrell et al., 2011; Matarazzo et al., 2012).

### Supplementary iron induces a metabolic shift toward a proteolytic and more toxic profile

The microbiome composition moved slightly toward a less favorable profile, but the most prominent effect under iron-rich conditions was the change of the microbial metabolome from a saccharolytic to a proteolytic profile. Lactate and formate levels were much lower in all conditions with supplementary iron compared to lowFe, also ethanol levels were decreased and succinate levels were decreased in the highFe conditions. These metabolites are primarily derived from carbohydrate, and levels of lactate and formate being the highest in the lowFe condition are consistent with previous findings of an *in vitro* study of Dostal et al. under extremely low iron conditions (Dostal et al., 2012b). Despite the apparent decrease in saccharolytic activity with iron in our model, the production of the mainly carbohydrate derived SCFAs was not decreased, probably due to the fact that they can also be produced from protein (Macfarlane and Macfarlane, 2012). More specifically, acetate and butyrate levels did not differ significantly compared to lowFe, but propionate levels were higher in the highFe conditions. This is partially in line with previous *in vivo* and *in vitro* findings of Dostal et al., although their data from different models were not always consistent at this point (Dostal et al., 2012a,b, 2014b).

In our model, the increased proteolytic activity under iron-rich conditions was evident by the increased levels of ammonia (a toxic metabolite), BCFAs and valerate, which are all protein fermentation-related metabolites (Smith and Macfarlane, 1997; Hughes et al., 2008; Hoyles and Wallace, 2010; Macfarlane and Macfarlane, 2012; Nery et al., 2012; Nyangale et al., 2012). The iron-induced switch toward a more proteolytic profile is further supported by the tendency that aromatic metabolites were increased. Importantly, it is well known that protein fermentation in general results in the formation of a putrefactive and toxic environment (Macfarlane and Macfarlane, 2012; Nyangale et al., 2012).

Our present study shows for the first time that the microbial metabolites that are produced under iron-supplemented (50FeS) conditions are indeed harmful to an *in vitro* monolayer of human intestinal epithelial cells. This was not evident from previous studies, but which may be explained by a different design and the use of different gut-model and cell-systems (Dostal et al., 2012b, 2014a).

### Supplementary iron induces an enrichment of certain virulence-associated pathways

Increased iron availability has been associated with an increase in virulence of bacterial pathogens (Bullen et al., 2005; Kortman et al., 2012). This inspired us to look into the abundance of virulence associated subsystems in our metagenome data. Flagella enable microbes to swim and swarm over surfaces and can be involved in adhesion to epithelial cells, which make them an important virulence factor (Moens and Vanderleyden, 1996). Intriguingly, our results show that the level-1 subsystem “Motility and Chemotaxis” was enriched in the 50FeS condition. Level-2 subsystems not only showed that flagella related subsystems were enriched in 50FeS, but also bacterial chemotaxis. Thus, iron could provide motile and chemotactic enteric pathogens with a competitive advantage over other members of the microbiota under iron-rich conditions.

### Possible explanations for the iron-induced shift in microbial metabolism

Our metabolome data represent a steady state situation that is the result of both metabolite production and consumption. Cross-feeding of microbial metabolites is anticipated to have a large influence on the metabolome composition (Nyangale et al., 2012; Rajilic-Stojanovic, 2013). Based on our 16S rDNA pyrosequencing and metagenomic data, and literature we can however provide specific leads for the explanation of the iron-induced changes in gut microbial metabolic activity. First of all, many metabolic pathways rely on the action of iron-dependent enzymes (ferredoxin-type enzymes with iron-sulfur clusters), for instance in the Wood-Ljungdahl pathway, and may thus be influenced by iron supplementation (Ragsdale and Pierce, 2008; Hood and Skaar, 2012). Secondly, redox balance needs to be maintained and microbial fermentation is regulated by this balance (Macfarlane and Macfarlane, 2003). It can be envisaged that supplementary iron has a large impact on the redox balance as this transition metal is able to function as an electron donor and electron acceptor. Importantly, also the availability of carbohydrates has a large impact on the type of metabolites that will ultimately be produced. Interestingly, when sufficient carbohydrate is present there is a decreased need to decarboxylate succinate to produce propionate, as has been observed for certain *Bacteroides* spp. (Macfarlane and Macfarlane, 2003). This fits with our findings that levels of succinate decreased while propionate levels increased in the high-iron conditions, indicating that carbohydrate sources were depleted early in the iron-rich conditions. Early depletion of carbohydrate sources by the gut microbiota in the conditions with supplementary iron may also clarify the increase in proteolytic activity. Furthermore, it may explain the low levels of lactate in the iron-supplemented conditions, but this might also be attributed to the lower abundance of lactic acid bacteria in the same conditions. An iron-induced increase in protein fermentation has previously also been observed in another *in vitro* colon model (Dostal et al., 2012b). This iron-induced shift toward a proteolytic profile has now been confirmed and studied in more detail. Carbohydrate substrates could have been limiting in the iron-supplemented conditions in the in *in vitro* models. It needs to be investigated whether carbohydrate sources become depleted early indeed, and whether this can also happen in the *in vivo* situation.

Although, the iron sources ferrous sulfate and ferric citrate differed in their counter-anion and initial valence state of the iron, they appeared not to cause marked differences in microbiome composition. However, differential results were observed on gut microbial activity and it appeared that the metabolic profile of the ferric citrate conditions was less proteolytic compared to the ferrous sulfate conditions. We however did not compare the *in vitro* cytotoxicity of the ferric citrate conditions with the ferrous sulfate conditions. Inorganic anions such as sulfate can affect microbial fermentation as these anions can function as an electron sink (Macfarlane and Macfarlane, 2003). Although the contribution of sulfate in the condition with ferrous sulfate was relatively small compared to the amount of sulfate already present in the SIEM and dialysis liquid (2.5–12.3% of the amount of sulfate present as magnesium sulfate), the additional sulfate may have influenced microbial metabolism. The citrate in the ferric citrate condition might have formed an alternative carbon source, but the contribution of citrate to the amount of carbon sources already present is also relatively small. Notwithstanding their biochemical differences, both ferrous sulfate and ferric citrate caused a shift toward a proteolytic profile.

Correlations of metabolite levels with the relative abundance of bacterial taxa do not provide a causal relationship but may provide some indication which taxa are responsible for the differences observed. It is interesting to see that *Prevotella*, whose growth was stimulated with iron, was positively correlated with propionate levels. *Prevotella* may drive the conversion of succinate to propionate (Nyangale et al., 2012), especially when carbon sources are limiting (as discussed above). From literature it is well-known that *Roseburia* and *Coprococcus* (both belong to the *Lachnospiraceae* family) and *Faecalibacterium* are producers of butyrate (Louis and Flint, 2009). Indeed we found a strong positive correlation of *Roseburia* and *Coprococcus* with butyrate levels, but butyrate levels do not correlate with *Faecalibacterium*. So far, very little is known about gut microbiota members that convert amino acids to BCFA. Suspected members are *Clostridium* spp. and *Atopobium*, but only *Clostridium coccoides* has previously been correlated with fecal BCFA levels (Thompson-Chagoyan et al., 2011; Rajilic-Stojanovic, 2013). Two groups of bacteria that are generally associated with proteolytic activity are *Bacteroides* and *Clostridium* (Cummings and Englyst, 1987; Nyangale et al., 2012), and although iron may theoretically have induced their metabolic activity, their abundance did not significantly change in our system. Candidate taxa that were identified in this study are *Lachnospiraceae, Oscillibacter, Clostridiales*, and *Desulfovibrionales* as they all positively correlated with BCFA and ammonia levels. Thus, *Lachnospiraceae* not only correlated with butyrate levels, but also strongly correlated with the BCFA and ammonia levels in our study, which has not been described before. Enrichment or depletion of certain genetic characteristics in the metagenomic data also provided clues about the microbial pathways involved in the iron-induced changes in gut microbial metabolism. The most obvious findings involved the protein- and amino acid related subsystems, such as “Protein Degradation,” “Ketoisovalerate Oxidoreductase,” “Aromatic Amino Acid Interconversions with Aryl Acids,” and “Aromatic Amin Catabolism” which were enriched in the 50FeS condition compared to the lowFe condition. It is interesting to note that the enzyme ketoisovalerate oxidoreductase needs iron as a cofactor, and is not only present in bacteria, but also in Archaea (Heider et al., 1996). This may indicate that also Archaea contributed to the iron-induced production of BCFAs.

### Supplementary iron decreases the abundance of siderophoric metabolites

Another intriguing aspect of our findings concerned the identification of the bacterial metabolites 2-hydroxy-isovalerate and 2-hydroxy-isocaproate that have siderophoric activity (Drechsel et al., 1993). The lowFe and hemin (an iron source that requires a different bacterial uptake mechanism Andrews et al., 2003) conditions had a lower total iron content compared to the mediumFe and highFe conditions. Indeed, levels of the iron-binding acids were relatively high in the lowFe and hemin conditions, while they were low or absent in the medium and high-iron conditions. This fits with the idea that bacteria only produce siderophores when readily available iron is scarce. We cannot be sure that these low affinity siderophoric metabolites indeed acted as siderophores, but as the levels of these aromatic amino acid-derived siderophores oppose the trend of the other aromatic amino acid-derived metabolites levels, our results suggest that limitation of iron may have triggered the production of these acids for at least a subset of the gut microbiota.

## Concluding remarks

In conclusion, this study shows that the provision of iron (provided as ferrous sulfate or as ferric citrate) to an *in vitro* human gut microbiota resulted in (i) a lower relative abundance of bacteria that are generally considered beneficial, (ii) an increase in toxic metabolites that impair the barrier function of cultured intestinal epithelial cells, (iii) and an increase in bacterial virulence-associated pathways. Thus, supplementation of these forms of iron creates a more hostile environment in the absence of host influences and under the conditions tested. The combination of these three effects could increase the risk for enteric infections. *In vivo*, the host immune status and the composition of the gut microbiota will largely determine whether or not an intestinal infection will become apparent. Our data also indicate that iron limitation increases the production of bacterial metabolites with iron-binding features. If and how these metabolites act as siderophores and mediate bacterial iron uptake under these conditions remains to be determined. One future challenge that remains, is to pinpoint metabolic changes to particular microbes, for which we provide novel leads in the present study. To generate better insight in the effects of iron on the actual microbial metabolism and to be able to pinpoint these metabolic properties to certain taxa, a metatranscriptomic approach is needed. This may be complemented with e.g., a LC-MS, HPLC, and/or NMR spectroscopy metabolomics approach to identify and quantify additional microbial volatile and non-volatile metabolites (Nyangale et al., 2012). Together this will help us in identifying microbial taxa that are responsible for the adverse effects in response to supplementary iron, and consequently, in developing an iron preparation that is highly available to humans, but has little to no adverse effects on the gut microbiota composition and metabolism. To reduce the risk for unwanted side effects this is an important challenge that remains in combating anemia in underdeveloped infection endemic regions.

## Materials and methods

### Origin of the microbiota

The microbiota for the TIM-2 experiments consisted of an active fecal microbiota of 6 Dutch adult volunteers [male: *n* = 3, age = 32.7 (21–39); female: *n* = 3, age = 33.3 (28–41)]. The healthy individuals were non-smokers and had not used antibiotics, prebiotics, probiotics or laxatives 3 months prior to the donation. Fecal samples were collected, kept in an anaerobic environment and were proceeded quickly to mixing and homogenization in an anaerobic cabinet. Mixed stools were used in a fed batch fermentor simulating the “cecum” conditions as described by Maathuis et al. (2012). Briefly, 670 ml medium without the addition of iron and hemin was inoculated with 80 g of mixed stools and incubated for 44 h at 37°C and under anaerobic conditions by a gaseous nitrogen flux, with fed-batch addition of 1250 ml medium over time. The composition of the microbiota after this batch fermentation was similar to the microbiota composition just after mixing the individual fecal samples (data not shown). Glycerol (10% final concentration) was added to the resulting fed-batch culture, that was aliquoted and snap-frozen in liquid nitrogen and stored at −80°C before inoculation in TIM-2. It has also previously been shown that these standardized samples are similar in composition and activity of a fresh fecal sample (Minekus et al., 1999; Venema et al., 2000; Kovatcheva-Datchary et al., 2009; Aguirre et al., 2015).

### Dynamic *in vitro* model of the large intestine (TIM-2)

TNO's *in vitro* model of the proximal large intestine (TIM-2) accurately simulates the average physical and dynamic conditions in the human proximal colon and has been described in detail before (Minekus et al., 1999; Maathuis et al., 2009; Rehman et al., 2012). TIM-2 is characterized by a physiological water content and constant removal of metabolites via a dialysis system, simulating host uptake, maintaining physiological levels of these metabolites, and allowing metabolite production kinetics to be recorded. This makes the model well suited to study both microbial composition and metabolic activity (Minekus et al., 1999; Venema et al., 2000). In short, the tightly computer controlled model consisted of glass units with a flexible wall inside through which peristaltic movements were achieved regularly. This way the lumen was mixed and transported through the system. Temperature was set at 37°C and the pH at 5.8 (simulating the pH in the proximal colon), controlled with a pH sensor in combination with secretion of sodium hydroxide. A dialysis system consisting of semipermeable hollow fibers was running through the lumen. A flow of dialysis liquid through the dialysis system constantly removed water and fermentation products. Therefore, physiological concentrations of small molecules, such as electrolytes, were retained and accumulation of microbial metabolites was prevented. A constant volume of the luminal content was maintained by water absorption controlled by a level sensor. The system was kept anaerobic by flushing with gaseous nitrogen. This resulted in the growth of a highly active and dense microbiota, previously shown to be comparable to that found in the human proximal colon (Minekus et al., 1999; Venema et al., 2000; Kovatcheva-Datchary et al., 2009).

### Standard ileal efflux medium (SIEM) and dialysis liquid composition

Standard ileal efflux medium (SIEM) was slightly modified for experiments in TIM-2 compared to the medium which was described by Gibson et al. (1988), mainly concerning a higher concentration of carbohydrates, pepton, casein and Tween 80 (Minekus et al., 1999; Van Nuenen et al., 2003). For the purpose of our experiments we removed the standard iron sources (0.009 g/L FeSO_4_.7H_2_O and 0.02 g hemin g/L) from the SIEM formulation to produce a non-iron supplemented lowFe control. Notably, it still contained low amounts of endogenous iron sources that come with the other ingredients. SIEM contained the following components (g/L): 9.0 pectin, 9.0 xylan, 9.0 arabinogalactan, 9.0 amylopectin, 43.7 casein, 74.6 starch, 31.5 Tween 80, 43.7 bactopepton, 0.7 ox-bile, 4.7 g K_2_HPO_4_.3H_2_O, 8.4 g NaCl, 0.7 g MgSO_4_.7H_2_O, 0.8 g CaCl_2_.2H_2_O, and 0.3 g cysteine.HCl, plus 1.5 ml of a vitamin mixture containing (per litre): 1 mg menadione, 2 mg D-biotin, 0.5 mg vitamin B12, 10 mg pantothenate, 5 mg nicotinamide, 5 mg p-aminobenzoic acid, and 4 mg thiamine. The pH was adjusted to 5.8. Low iron dialysis liquid contained (per litre): 2.5 g K_2_HPO_4_.3H_2_O, 4.5 g NaCl, 0.5 g MgSO_4_.7H_2_O, 0.45 g CaCl_2_.2H_2_O, 0.05 g bile, and 0.4 g cysteine.HCl, plus 1 ml of a vitamin mixture as mentioned above. The pH was adjusted to 5.8. All medium components were provided by Tritium Microbiology (Eindhoven, The Netherlands). SIEM did not require pre-digestion. For the experiments the iron sources as described below were added to the SIEM and dialysis liquid.

### TIM-2 experiments and test conditions

**Figure 8:**
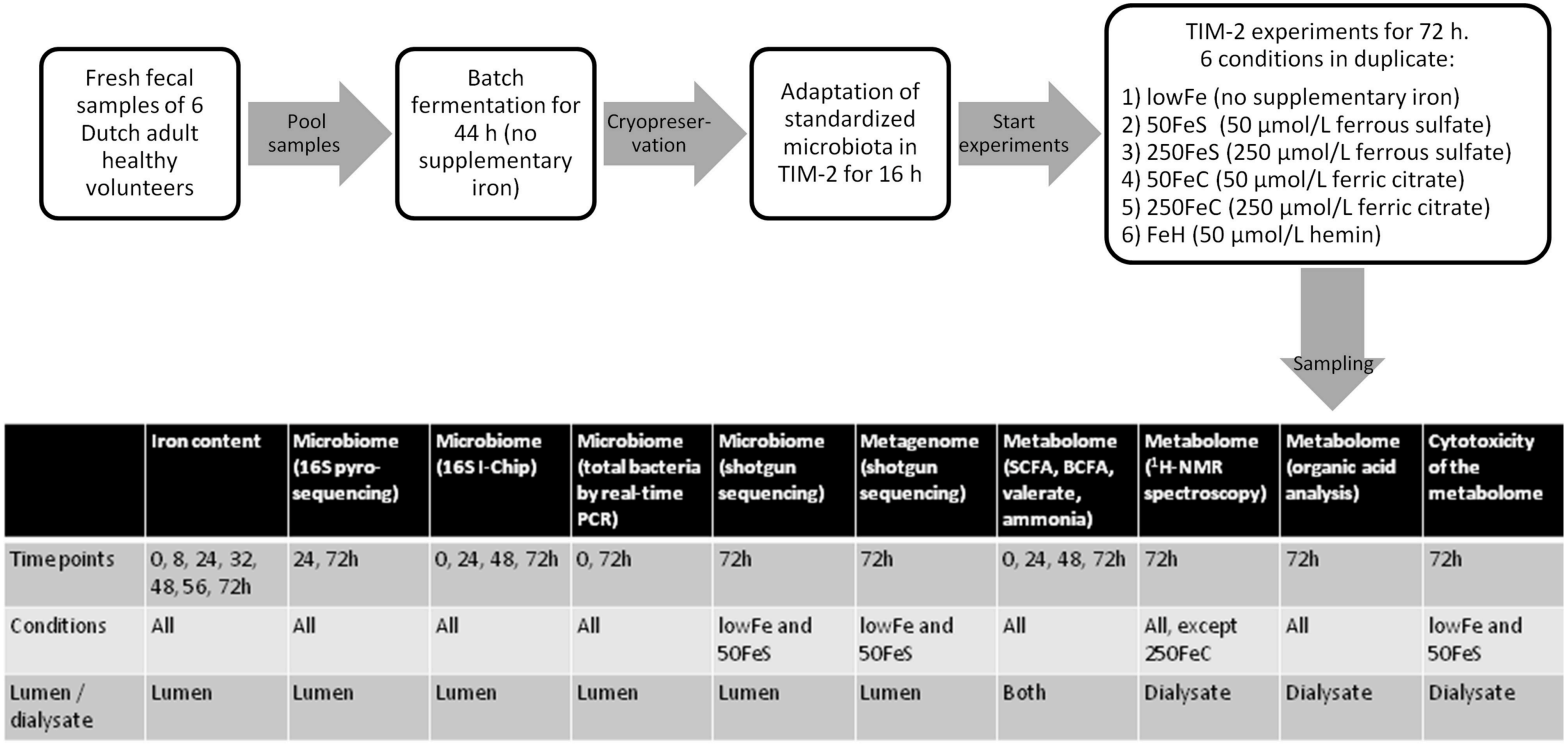
**Scheme of the experimental setup and overview of sample analysis**. Experimental setup and workflow of the TIM-2 experiments, and a table that shows what type of analyses were performed on which samples.

Experiments were carried out as described before (Maathuis et al., 2012). At the start of each experiment, the model was inoculated with approximately 30 mL of the standardized microbiota (that was used for each experiment, in duplicate) and 80 mL of dialysate. The microbiota was allowed to adapt to the model conditions for 16 h. Then a 3-day experimental period was started. Interventions with iron were as follows: (i) no additional iron (lowFe), (ii) 50 μmol/L ferrous sulfate (50FeS), (iii) 250 μmol/L ferrous sulfate (250FeS), (iv) 50 μmol/L ferric citrate (50FeC), (v) 250 μmol/L ferric citrate (250FeC), and (vi) 50 μmol/L hemin (FeH). The experiments with addition of ferrous sulfate and ferric citrate were designed to mimic oral iron supplementation in medium and high amounts, and also to investigate whether these different iron sources have differential effects on microbial composition and metabolism. Although hemin will not be used as an oral iron supplement, we included this iron source as an additional condition to investigate how the gut microbiota responds to an iron source that that requires a different uptake mechanism. Iron was added to the lumen at *t* = 0, 24, and 48 h and was also provided in the constant flow of dialysis liquid to prevent loss of iron from the lumen. While the lowFe condition can be regarded as mildly iron-deficient, the mediumFe and highFe can be best described as iron-supplemented conditions. The effective iron content of the TIM-2 lumen during our experiments, compared to *in situ* iron content of human feces, is shown in Supplementary Data 1. Samples were taken from the lumen and from the dialysate starting after the adaptation period (set as time point 0) and at 8, 24, 32, 48, 56, and 72 h after starting the iron intervention. These were used to determine microbiota composition, levels of iron, SCFA, BCFA, ammonia, and to profile the metabolome by ^1^H-NMR spectroscopy and GC-MS organic acid analysis. After 24 and 48 h, a total lumen sample of 25 mL was removed from the system to simulate passage of material from the proximal to the distal colon. This way a physiological colonic transit time of 48 h was mimicked. In each experiment, SIEM was fed during the 3-day experimental period with 60 mL/day and contained the iron source and concentration corresponding to the applied conditions. Figure 8 shows the experimental scheme and summarizes the analyses there were done on the various samples.

### SCFA/BCFA quantification in lumen and dialysate samples

Samples (*t* = 0, 24, 48, and 72 h) were centrifuged for 5 min at 12,000 g and a mixture of formic acid (20%), methanol and 2-ethyl butyric acid (internal standard, 2 mg/ml in methanol) was added to the clear supernatant. A 0.5 μl sample was injected on a GC-column (Stabilwax-DA, length 15 m, ID 0.53 mm, film thickness 0.1 mm; Varian Chrompack, The Netherlands) in a Chrompack CP9001 gas chromatograph using an automatic sampler (Chrompack liquid sampler CP9050; Varian Chrompack). Specifically, concentrations of acetate, propionate, butyrate, valerate, isovalerate, and isobutyrate were determined (Maathuis et al., 2009).

### Ammonia quantification in lumen and dialysate samples

Samples (*t* = 0, 24, 48, and 72 h) were centrifuged for 5 min at 12,000 g. Ammonia in the supernatant was determined based on the Berthelot reaction, in which ammonia first reacts with alkaline phenol and then with sodium hypochlorite to form indophenol blue. The absorbance of the indophenol blue is directly proportional to the original ammonia concentration and is measured at 660 nm. The measurement was automated on a Cobas Mira Plus autoanalyzer (Roche, Almere, The Netherlands) and was performed by Bio-aNAlytiX (Mook, the Netherlands). Concentrations in the samples were determined via comparison with a series of standard solutions with known concentrations (Maathuis et al., 2009).

### ^1^H-NMR spectroscopy of dialysate samples

Dialysate samples of lowFe, 50FeS, 250FeS, FeH and 50FeC at 72 h were measured with 1D and 2D COSY NMR spectroscopy. 250FeC was not analyzed because of expected interference of large amounts of H in the citrate. A small volume (20 μl) of 20.2 mM trimethylsilyl-2,2,3,3-tetradeutero-propionic acid (TSP, sodium salt; Aldrich) in D_2_O was added to 700 μl of the sample, providing a chemical shift reference (0.00 ppm) and a deuterium lock signal. To aid the identification of metabolites, the pH was adjusted to 2.50 and 7.00 using concentrated HCl. Finally, 650 μl of the sample was placed in a 5 mm NMR tube (Wilmad Royal Imperial; Wilmad LabGlass, USA). ^1^H-NMR spectra were obtained on a Bruker Avance I 500 spectrometer, operating at 11.7 T, with a Broad Band Inverse (BBI) probehead equipped with a actively shielded z-gradient coil. 1D ^1^H spectra were acquired as 256 transients in 64K data points with a spectral width of 10080 Hz. The sample temperature was 298K; the H_2_O resonance was presaturated by single-frequency irradiation during a relaxation delay of 4 s; a pulse width of 7 μs was used (corresponded to a 90° excitation pulse). Shimming of the sample was performed automatically using the Topshim shim program (Bruker). The resonance line widths for TSP and all metabolites were <1 Hz. Phase and baseline were corrected manually. All spectra were scaled to TSP and metabolite and TSP resonances were fitted semi-automatically with a Lorentzian line shape. Metabolite concentrations in the samples (pH 2.5) were calculated to the known concentration of the TSP standard (singlet, 9 protons) and expressed as mmol/l. Resonances were assigned by comparison the measured data with our model compound database containing relevant compounds (U. Engelke, unpublished results) and the commercial available metabolite database from Bruker (bbiorefcode-2-0-0).In the 2D COSY spectra, the spectral widths in the F1 and F2 domains were 6002 Hz. A total of 4K data points were collected in t2, 128 t1 increments with 16 transients per increment were used, and the relaxation delay was set to 2 s. Before the Fourier transformation, a sine-bell function was applied in both time domains. During the relaxation delay, the water resonance was pre-saturated.

### Organic acid analysis of dialysate by GC-MS

Organic acids were analyzed using standard techniques. Briefly, to 0.5 mL of dialysate sample (72 h) 2.5 mL SETH buffer [0.25 mol/L sucrose; 2 mmol/L (K)EDTA; 10 mmol/L Tris; 5 × 10^4^ U heparin (pH 7.4)] was added. The mixture was acidified to pH2 with concentrated HCl (10%), after which the organic acids were extracted by ethylacetate twice, derivatized with trimethylsilyl (TMS), and analyzed on an Agilent 7890A gas chromatograph (GC), coupled to a flame ionization detector (FID) and an Agilent 5975C inert XL MSD mass spectrometer. Quantification of organic acids was done by calculation of peak areas and comparison with an internal standard (4-phenylbutyric acid). Relative concentrations were expressed relative to the lowFe condition which was set at 100%.

### Analysis of gut microbial composition, functions, and metabolome toxicity

Full descriptions of the methods used to determine luminal iron content, microbiome composition, gut microbial functions, and fecal water toxicity, are available in the online Supplementary Materials and Methods.

### Statistics and data presentation

#### Statistical analysis of total bacteria qPCR data, metabolic data, and cell cytotoxicity data

TIM-2 experiments were performed in duplicate (*n* = 2) which is a small sample size, but we have previously shown that the variations in the system are small because of the fact that a standardized microbiota is used and because the system is computer controlled (Venema et al., 2000). Total bacteria were compared between time points by a paired *t*-test and among the single iron conditions by a Two-way-ANOVA with repeated measures and Bonferroni's *post-hoc* test. The effects of iron on the metabolome were compared among the single conditions, but also as pooled data (when appropriate); mediumFe (50 μmol/L ferrous sulfate and 50 μmol/L ferric citrate; *n* = 4) and highFe (250 μmol/L ferrous sulfate and 250 μmol/L ferric citrate; *n* = 4). Data from the hemin condition, a different type of iron source, was never pooled with another condition. When two doses of the same iron source were pooled this is indicated as FeS (both ferrous sulfate conditions; *n* = 4) and FeC (both ferric citrate conditions; *n* = 4). Metabolite levels, presented as mean + range, were compared by Tukey's multiple comparison *post-hoc* test. Cytotoxicity of dialysates to Caco-2 monolayers was tested at least in triplicate and results were expressed as mean + SD. The course of TEER was compared by linear regression analysis of the slope. Means of phenol red permeability, LDH-release and trypan blue staining data were compared by an unpaired *t*-test. In case of unequal variances (as assessed by F-test) an unpaired *t*-test with Welch's correction was carried out. Analysis was performed using GraphPad Prism version 5.03 for Windows, GraphPad Software, San Diego California USA. *P*-values < 0.05 were considered statistically significant and *P*-values < 0.10 were considered as an important trend throughout the paper.

#### Analysis of 16S rDNA sequencing data, microarray (I-Chip) data, and metagenomic sequencing data

Full descriptions are available in the online supplementary materials and methods.

## Data access

The sequencing datasets accompanying this study are available in the European Nucleotide Archive with the study accession number [ENA: PRJEB6542].

## Author contributions

GK participated in the design and coordination of the study, performed iron measurements, cell cytotoxicity experiments, analyzed and interpreted data, and drafted the manuscript. BD analyzed and interpreted I-Chip and metagenomic sequencing data, and helped to draft the manuscript. AM participated in the design and coordination of the study and performed the TIM-2 experiments, including total bacteria qPCR, and measurement of ammonia and short chain fatty acids, and helped to draft the manuscript. UE performed ^1^H-NMR spectroscopy and analysis, and helped to draft the manuscript. JB(1) analyzed 16S rRNA pyrosequencing data. KK helped to analyze I-Chip data and critically revised the manuscript. FN, JB(2), JW and ZK participated in the shotgun metagenomic sequencing. LK performed organic acid analysis and helped to draft the manuscript. DS interpreted data and critically revised the manuscript. KV and HT were involved in the design and coordination of study, interpreted data, and helped to draft the manuscript. All authors read and approved the final manuscript.

## Funding

This work was in part supported by the Dutch Digestive Diseases Foundation (project WO 10-53). BD was supported by the Netherlands Organization for Scientific Research (NWO) Vidi grant 864.14.004, the Dutch Virgo Consortium (FES0908, NGI 050-060-452), and CAPES/BRASIL.

### Conflict of interest statement

The authors declare that the research was conducted in the absence of any commercial or financial relationships that could be construed as a potential conflict of interest.
