# Supplementary material for: Microbial Metabolism Shifts Towards an Adverse Profile with Supplementary Iron in the TIM-2 In vitro Model of the Human Colon
Source: Front Microbiol. 2016 Jan 6;6:1481. doi: 10.3389/fmicb.2015.01481 (PMC4701948; doi:10.3389/fmicb.2015.01481)
Supplement: Supplementary file 1 [file DataSheet1.zip › Supplementary material/Supplementary Data 1.pdf]

# Microbial metabolism shifts towards an adverse profile with supplementary iron in the TIM-2 *in vitro* model of the human colon

Guus AM. Kortman, Bas E. Dutilh, Annet JH. Maathuis, Udo F. Engelke, Jos Boekhorst, Kevin P. Keegan, Fiona Nielsen, Jason Betley, Jacqueline Weir, Zoya Kingsbury, Leo AJ. Kluijtmans, Dorine W. Swinkels, Koen Venema, Harold Tjalsma.

## Supplementary Data 1

**Total iron concentration in the TIM-2 lumen**

The total iron content of the TIM-2 lumen was determined over time to study the effect of the different iron sources, iron dose and our dosing schedule on the actual luminal iron content. This also allowed us to compare TIM-2 luminal iron content with the *in situ* iron content of human fecal samples. The iron content of the non-supplemented lowFe (see materials and methods) condition on average (range 8 – 72h) was 1.0 (0.7 – 1.3) mg/L and did not increase over time. Conversely, the iron concentration markedly increased over time in all conditions with supplementary iron (**Supplementary Figure 1)**. For the 50FeS and 250FeS conditions (50 and 250 µmol/L ferrous sulfate) the average concentrations were 10.4 (3.7 – 15.4) and 24.3 (9.6 – 34.8) mg/L respectively. In the 50FeC and 250FeC conditions (50 and 250 µmol/L ferric citrate) it was 8.4 (2.8 – 12.7) and 21.0 (10.3 – 32.4) mg/L respectively. In FeH (50 µmol/L hemin; that requires different bacterial uptake systems) the average concentration was 4.2 (1.8 – 6.9) mg/L. The iron concentration in feces of Dutch adult volunteers, extrapolated to the higher water content of the standard ileal efflux medium (SIEM; the water content of SIEM is approximately three times higher than in human feces), was estimated to be 19.5±3.5 µmol/L (**Supplementary Figure 1)**. Thus, compared to the total iron concentration in feces, the iron content of the lowFe condition can be described as mildly iron-deficient.

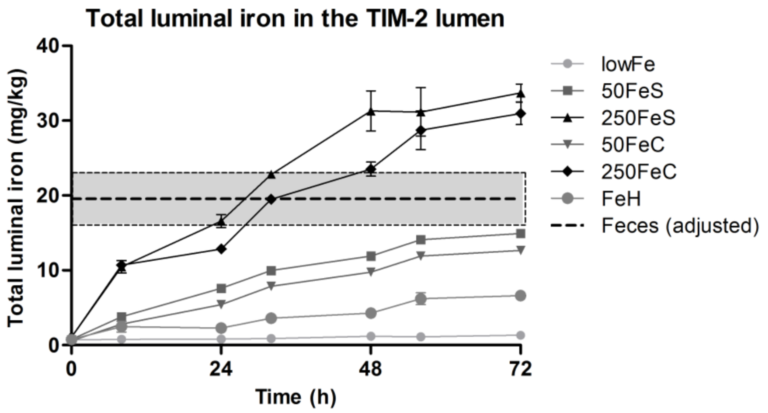


**Supplementary Figure 1. Iron content of the TIM-2 lumen in the various iron conditions**
Average total iron concentration in the TIM-2 lumen over time (mean±range;n = 2 for each time point). Supplementary iron accumulated in the lumen during the 72h incubation. As a reference, the total iron content measured in feces of Dutch adult volunteers (n=3) is indicated by the dashed line (mean±SD). The feces was diluted three times to obtain a similar consistency as the TIM-2 lumen which has a higher water content as compared to human feces.

It should be noted that the majority of the supplemented iron was bound to solid matter of the SIEM, or was precipitated in the TIM-2 lumen, and that iron concentration in the 'fecal water' (lumen supernatant) was much lower and relatively stable over time compared to the total lumen sample (data not shown). Iron could accumulate via influx of iron from the dialysis liquid and feeding the system with fresh SIEM. The fecal iron concentration of 59 mg Fe/kg wet weight feces (undiluted) as measured by an iron-releasing and chromogenic method, reflected the fecal iron content of healthy Dutch human adults. This is somewhat lower compared to the approximate 100 mg Fe/kg wet weight feces that was found in British adults in a study of Lund *et al.* (Lund et al., 1999). Remarkably, Chile infants fed with unfortified cow's milk formula had an average fecal iron content of 60 mg Fe/kg, which is higher than expected for such a diet and a group with an iron deficiency prevalence of 25.7%. The iron content even increased to 100 mg Fe/kg feces when solid foods were introduced at the age of 7 months (Pizarro et al., 1987). Based on these studies our highFe condition would reflect a rather normal iron content, even when compared to the fecal iron content of infants. However, iron supplemented as ferrous sulfate or ferric citrate is very different from less readily available endogenous iron sources such as present in grains and vegetables. Therefore our mediumFe and highFe conditions can be best described as iron-supplemented conditions. Readily (freely) available iron concentrations and iron speciation in the colon lumen remain however very difficult to predict and to measure.

## References

Lund, E.K., Wharf, S.G., Fairweather-Tait, S.J., and Johnson, I.T. (1999). Oral ferrous sulfate supplements increase the free radical-generating capacity of feces from healthy volunteers. *Am J Clin Nutr* 69**,** 250-255.

Pizarro, F., Amar, M., and Stekel, A. (1987). Determination of iron in stools as a method to monitor consumption of iron-fortified products in infants. *Am J Clin Nutr* 45**,** 484-487.
