# Supplementary material for: Microbial Metabolism Shifts Towards an Adverse Profile with Supplementary Iron in the TIM-2 In vitro Model of the Human Colon
Source: Front Microbiol. 2016 Jan 6;6:1481. doi: 10.3389/fmicb.2015.01481 (PMC4701948; doi:10.3389/fmicb.2015.01481)
Supplement: Supplementary file 1 [file DataSheet1.zip › Supplementary material/Supplementary Data 2 .pdf]

Microbial metabolism shifts towards an adverse profile with supplementary iron in the TIM-2 *in vitro* model of the human colon

Guus AM. Kortman, Bas E. Dutilh, Annet JH. Maathuis, Udo F. Engelke, Jos Boekhorst, Kevin P. Keegan, Fiona Nielsen, Jason Betley, Jacqueline Weir, Zoya Kingsbury, Leo AJ. Kluijtmans, Dorine W. Swinkels, Koen Venema, Harold Tjalsma.

Supplementary Data 2

Total bacteria content of the lumen

To study the effect of the various iron sources and concentrations on the total number of bacteria in the lumen of TIM-2, the number of bacteria was determined at t = 0h and 72h by qPCR. Overall, the number of bacteria was higher at 72h compared to 0h ($p = 0.045$), but iron source or concentration had no significant effect on the outcome ($p = 0.14$). Comparisons within the single conditions showed that the total number of bacteria was significantly increased from 0 to 72h in 50FeC only ($p < 0.01$) (**Supplementary Figure 2**). The microbiota density may appear low, but it should be taken into account that the TIM-2 model simulates the average conditions in the human proximal colon, where much more food and especially water is present compared to faeces.

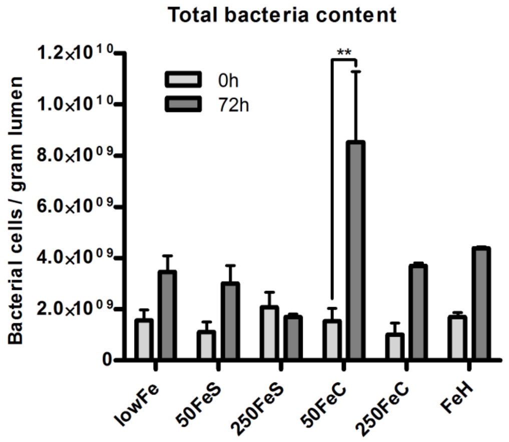

Supplementary Figure 2. Total bacterial cells in the TIM-2 lumen at baseline and at 72h
Total number of bacteria (mean+range; n = 2) as determined by qPCR in the TIM-2 lumen was similar at 0h (baseline) and was generally increased at 72h ($p = 0.045$). This increase was significant for the 50FeC condition only when the single conditions were analyzed. Overall, iron source or concentration had no effect on the outcome on the change in total bacteria from baseline to 72h. ** $p < 0.01$.

Microbiome analysis by 16S rRNA sequencing

We determined the microbiome composition by analyzing a total of 82,681 bacterial 16S rRNA sequences obtained by pyrosequencing. Overall, the microbiome (samples taken at 24h and 72h combined) consisted of the phyla Firmicutes (58.4% of the 16S rRNA reads), Bacteroidetes (33.8%), Actinobacteria (6.9%) and Proteobacteria (0.8%) (**Supplementary Figure 3**). At 24h, the phylogenetic diversity index (a measure of α-diversity) was similar among all conditions and was not yet affected by iron. However, at 72h the diversity was different among groups ($p = 0.046$), where the diversity of the lowFe condition was lower compared to the highFe (250FeS and 250FeC combined) conditions ($p < 0.1$) (**Supplementary Figure 4A**). Supplementation of iron as ferrous sulfate (FeS) or

ferric citrate (FeC) did not have a significant differential effect on diversity (**Supplementary Figure**

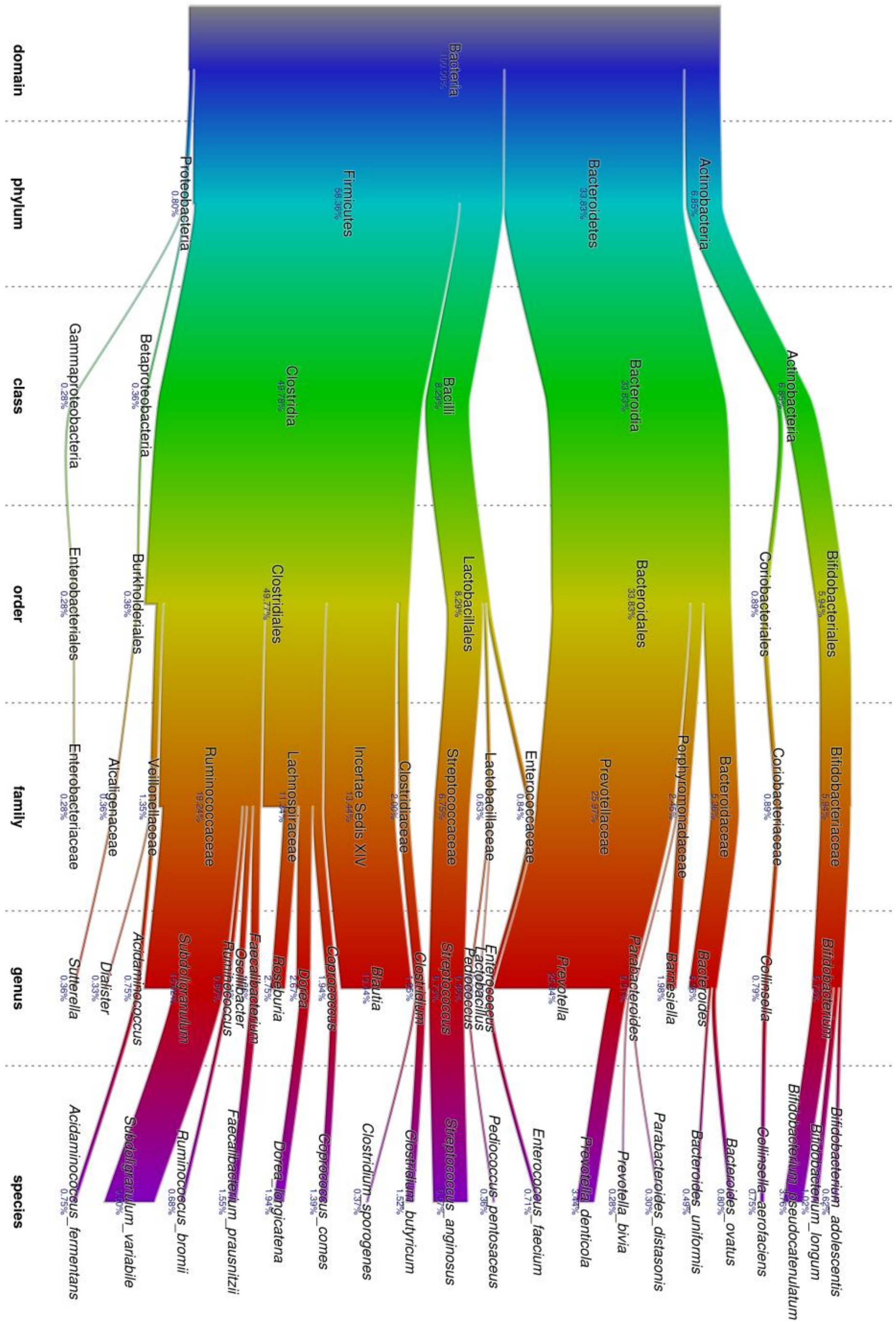

4B).

Supplementary Figure 3. Overall gut microbiome as determined by 16S rRNA pyrosequencing

This visualization shows the average abundance (in %) of 16S rRNA reads attributed to the specific taxonomic level and represents all samples in the 16S rRNA pyrosequencing analysis (all conditions at 24h and at 72h; n=24).

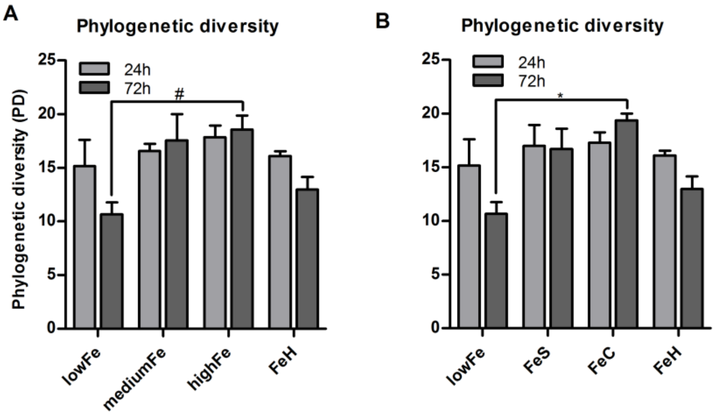

Supplementary Figure 4. Effect of iron on the phylogenetic diversity

The phylogenetic diversity (PD) index of the lowFe, mediumFe (pooled 50FeS and 50FeC conditions), highFe (pooled 250FeS and 250FeC conditions), FeH (50 µmol/L hemin) is given at 24h and 72h (median + range; n = 2-4). At 24h medians were similar, but at 72h medians differed significantly (P = 0.046) and the diversity of lowFe was lower compared to highFe (**A**).The phylogenetic diversity (PD) index of the lowFe, FeS (pooled 50FeS and 250FeS conditions), FeC (pooled 50FeC and 250FeC conditions), and FeH at 24h and 72h (median + range; n = 2) is given in panel B. At 72h, medians tended to differ significantly (P = 0.023), and the diversity of lowFe was lower compared to FeC (**B**). For this analysis the number of reads per sample was downsampled to 1930.
