# Supplementary material for: Microbial Metabolism Shifts Towards an Adverse Profile with Supplementary Iron in the TIM-2 In vitro Model of the Human Colon
Source: Front Microbiol. 2016 Jan 6;6:1481. doi: 10.3389/fmicb.2015.01481 (PMC4701948; doi:10.3389/fmicb.2015.01481)
Supplement: Supplementary file 1 [file DataSheet1.zip › Supplementary material/Supplementary Figure 5-10.pdf]

Microbial metabolism shifts towards an adverse profile with supplementary iron in the TIM-2 *in vitro* model of the human colon

Guus AM. Kortman, Bas E. Dutilh, Annet JH. Maathuis, Udo F. Engelke, Jos Boekhorst, Kevin P. Keegan, Fiona Nielsen, Jason Betley, Jacqueline Weir, Zoya Kingsbury, Leo AJ. Kluijtmans, Dorine W. Swinkels, Koen Venema, Harold Tjalsma.

Supplementary Figures 5-10

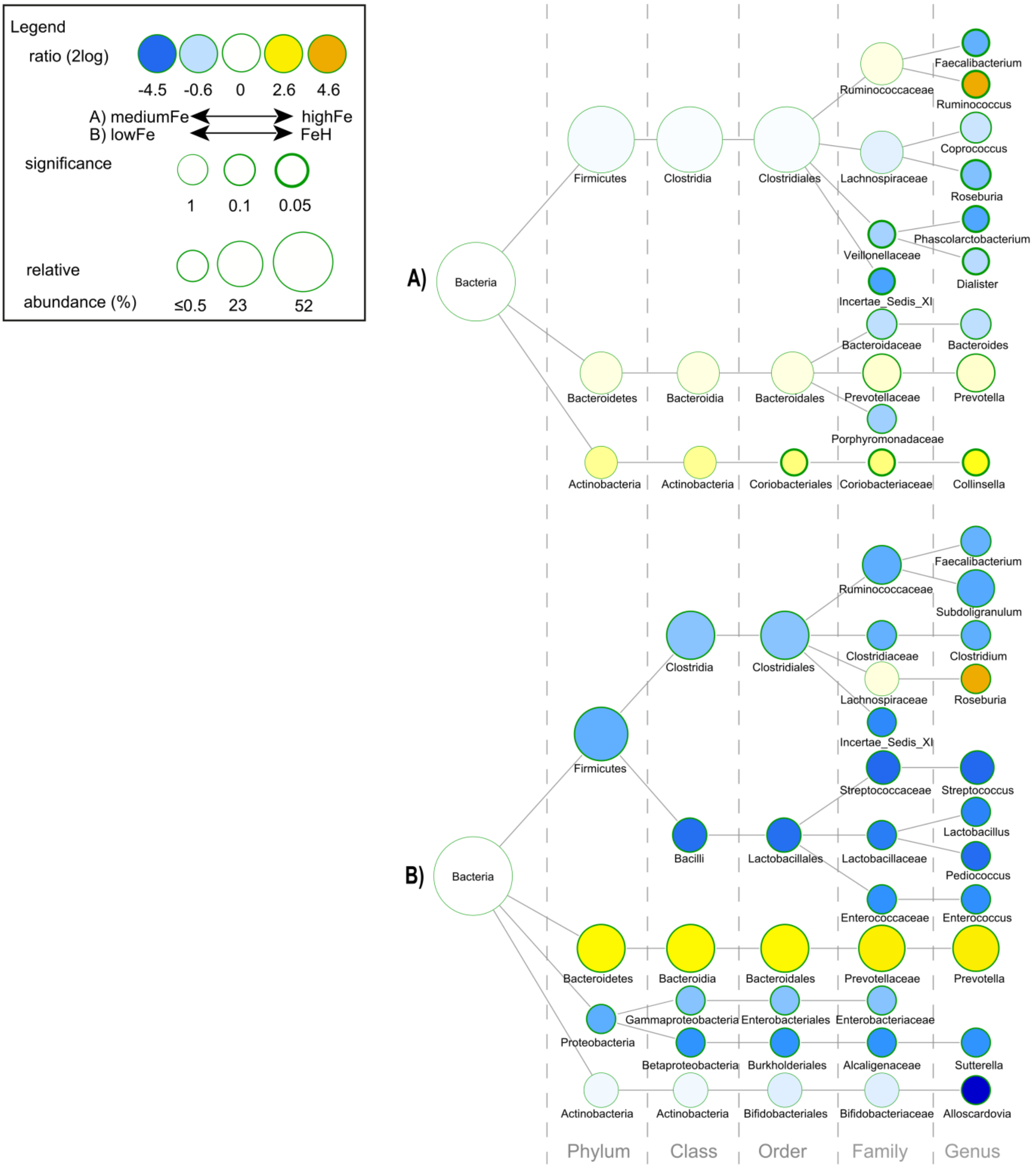

Supplementary Figure 5. Differences in gut microbial composition between the mediumFe and the highFe conditions and differences between the lowFe and FeH condition at 72h

Nodes represent taxa; edges link the different taxonomic levels. The fold difference is calculated as the 2log of the ratio of the relative abundance in the mediumFe (n=4) and highFe (n=4) conditions (**A**) or as the 2log ratio of the relative abundance in the lowFe (n=2) and FeH (n=2) conditions (**B**) (0 = no difference between groups, 1 = twice as abundant in mediumFe/highFe, etc.). In this explorative analysis, the significance is expressed as the p value of a Mann-Whitney U test. The node-size corresponds to the relative abundance. Taxa (that is, nodes) were included in this visualization when the fold difference met a significance level of $p < 0.1$, or when the taxon had a child (that is, more specific taxonomic classification) meeting this criterion.

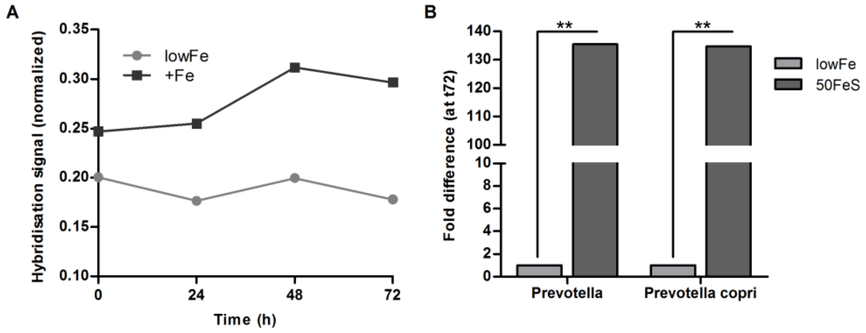

Supplementary Figure 6. The effect of supplementary iron on *Prevotella* abundance

I-Chip analysis showed that the *Prevotella* level (all *Prevotella* probes on the chip taken together; n=9) was fairly constant in the lowFe condition, while there was a visible increase over time in the conditions with iron (pool of all iron conditions; +Fe) (**A**). Metagenomic analysis showed a large fold difference in genus *Prevotella* at 72h between the lowFe (set to 1) and 50FeS condition (2log ratio: 7.07; p = 0.006) (both n=2). For the majority this appeared to be attributable to the species *Prevotella copri* (p = 0.006), but which was also the only *Prevotella* species that was found in the metagenomic database that we used for annotation (Qin et al., 2010) (**B**).

Interestingly, we here show that supplementary iron increased the abundance of *Prevotella*, while a relatively high *Prevotella* abundance has been associated with carbohydrate rich plant based diets and has been found to be more abundant in African children compared to European children (De Filippo et al., 2010;Wu et al., 2011;Yatsunenko et al., 2012). It is however difficult to speculate how these finding may relate to the iron-induced increase in *Prevotella* in TIM-2.

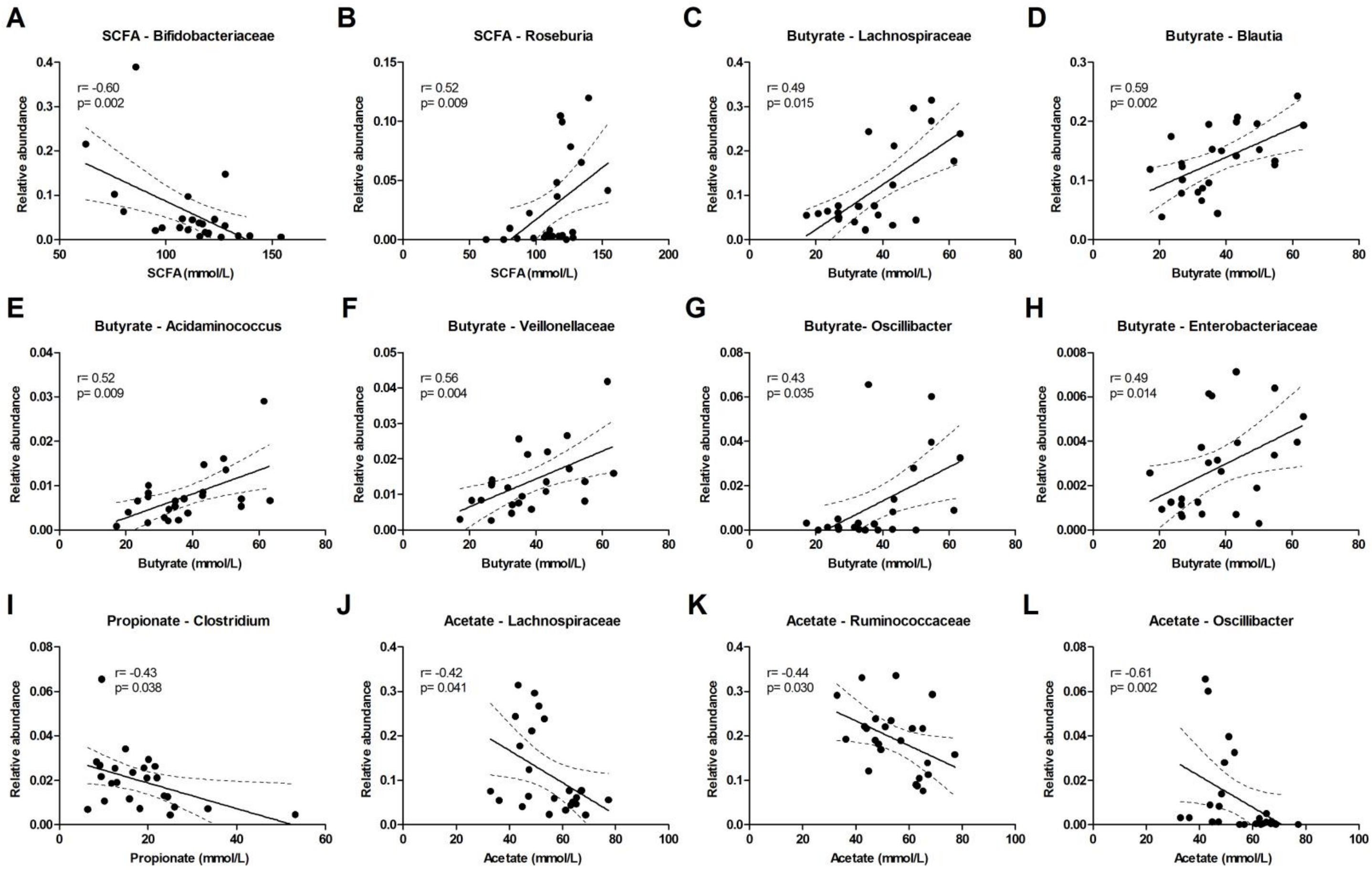

Supplementary Figure 7. Overview of additional bacterial taxa that correlate with luminal SCFA, acetate, butyrate and propionate levels

The relative abundance (fraction of the total 16S rRNA) of bacterial taxa in all samples at 24h and 72h (n=24) (as determined by 16S rRNA pyrosequencing) was correlated with luminal SCFA (**A-B**), butyrate (**C-H**) propionate (**I**) and acetate (**J-L**) levels in the same samples. Best fit lines with 95% confidence bands were generated by linear regression analysis. Spearman r and p-value are displayed for each graph.

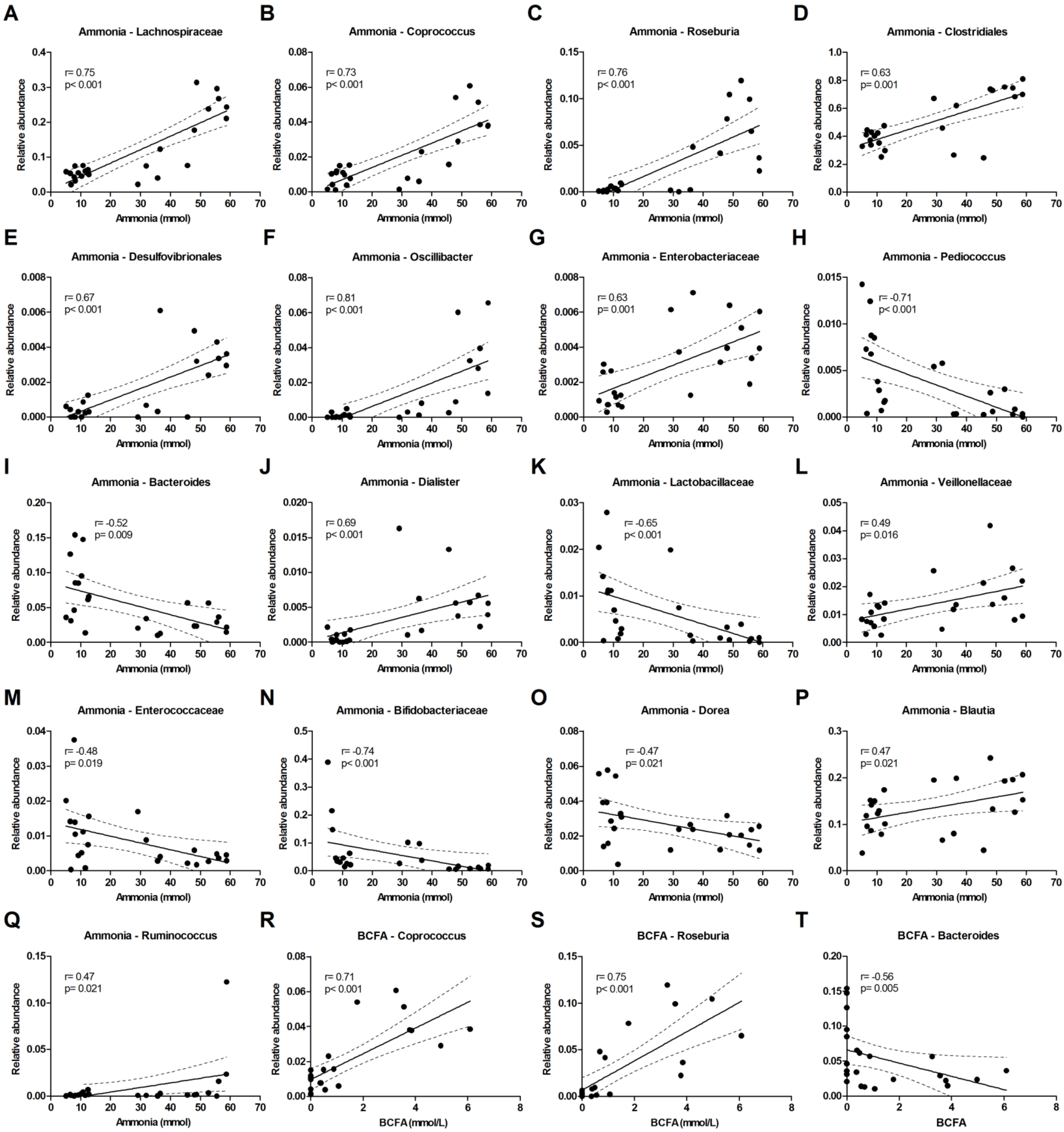

Supplementary Figure 8. Overview of bacterial taxa that correlate with cumulative ammonia levels and additional taxa that correlate with luminal BCFA levels

The relative abundance (fraction of the total 16S rRNA) of bacterial taxa in all samples at 24h and 72h (n=24) (as determined by 16S rRNA pyrosequencing) was correlated with luminal ammonia levels in the same samples at 24h and cumulative ammonia levels at 72h (**A-Q**). Panels **R-T** show additional (to the main article) correlations of the relative abundance of bacterial taxa at 24h and 72h with luminal BCFA levels in the same samples. Best fit lines with 95% confidence bands were generated by linear regression analysis. Spearman r and p-value are displayed for each graph.

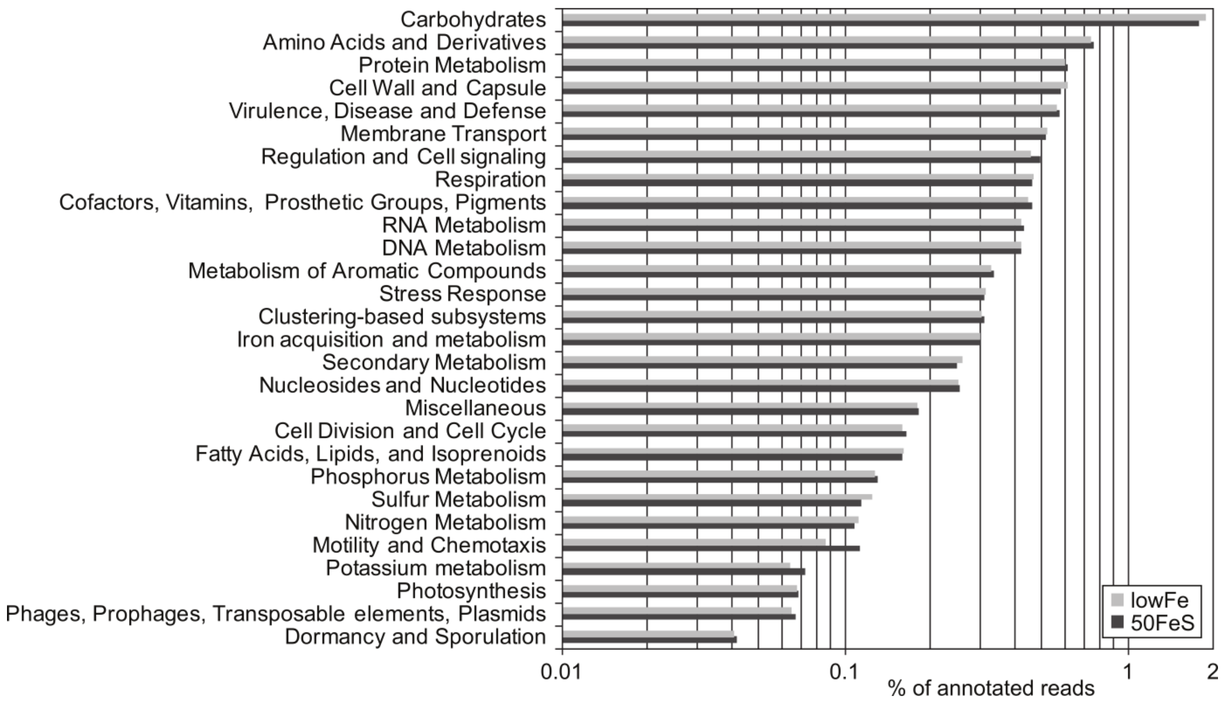

Supplementary Figure 9. Functional analysis of metagenomic sequencing data

Overview of the functions that were present in the lowFe and 50FeS conditions (both n=2) (Level-1 SEED subsystems) and the proportion of reads that were annotated to these functions.

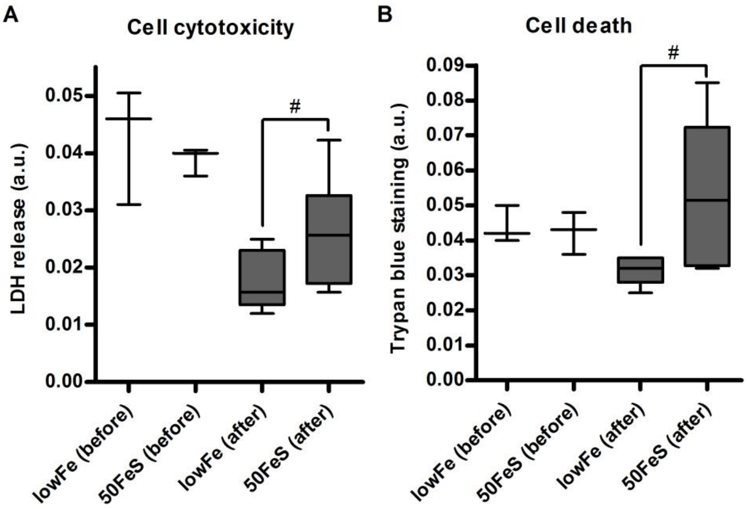

Supplementary Figure 10. Effect of iron on the microbial metabolome toxicity towards a Caco-2 intestinal monolayer

Effect of dialysis liquids (before exchange in TIM-2; not containing microbial metabolites) and dialysates (after exchange in TIM-2; at 72h) of the lowFe condition and 50FeS condition on cell cytotoxicity and cell death of a Caco-2 monolayer. Cell cytotoxicity as determined by LDH-release (whiskers min to max) tended to be higher in the 50FeS dialysate compared to the lowFe dialysate (n=6) (**A**). Cell death as determined by trypan blue staining (whiskers min to max) tended to be higher in the 50FeS dialysate compared to the lowFe dialysate (n=6) (**B**). # $p < 0.1$.

References

De Filippo, C., Cavalieri, D., Di Paola, M., Ramazzotti, M., Poullet, J.B., Massart, S., Collini, S., Pieraccini, G., and Lionetti, P. (2010). Impact of diet in shaping gut microbiota revealed by a comparative study in children from Europe and rural Africa. *Proc Natl Acad Sci U S A* 107**,** 14691-14696.

Qin, J., Li, R., Raes, J., Arumugam, M., Burgdorf, K.S., Manichanh, C., Nielsen, T., Pons, N., Levenez, F., Yamada, T., Mende, D.R., Li, J., Xu, J., Li, S., Li, D., Cao, J., Wang, B., Liang, H., Zheng, H., Xie, Y., Tap, J., Lepage, P., Bertalan, M., Batto, J.M., Hansen, T., Le Paslier, D., Linneberg, A., Nielsen, H.B., Pelletier, E., Renault, P., Sicheritz-Ponten, T., Turner, K., Zhu, H., Yu, C., Li, S., Jian, M., Zhou, Y., Li, Y., Zhang, X., Li, S., Qin, N., Yang, H., Wang, J., Brunak, S., Dore, J., Guarner, F., Kristiansen, K., Pedersen, O., Parkhill, J., Weissenbach, J., Bork, P., Ehrlich, S.D., and Wang, J. (2010). A human gut microbial gene catalogue established by metagenomic sequencing. *Nature* 464**,** 59-65.

Wu, G.D., Chen, J., Hoffmann, C., Bittinger, K., Chen, Y.Y., Keilbaugh, S.A., Bewtra, M., Knights, D., Walters, W.A., Knight, R., Sinha, R., Gilroy, E., Gupta, K., Baldassano, R., Nessel, L., Li, H., Bushman, F.D., and Lewis, J.D. (2011). Linking long-term dietary patterns with gut microbial enterotypes. *Science* 334**,** 105-108.

Yatsunenko, T., Rey, F.E., Manary, M.J., Trehan, I., Dominguez-Bello, M.G., Contreras, M., Magris, M., Hidalgo, G., Baldassano, R.N., Anokhin, A.P., Heath, A.C., Warner, B., Reeder, J., Kuczynski, J., Caporaso, J.G., Lozupone, C.A., Lauber, C., Clemente, J.C., Knights, D., Knight, R., and Gordon, J.I. (2012). Human gut microbiome viewed across age and geography. *Nature* 486**,** 222-227.
