# Supplementary material for: Microbial Metabolism Shifts Towards an Adverse Profile with Supplementary Iron in the TIM-2 In vitro Model of the Human Colon
Source: Front Microbiol. 2016 Jan 6;6:1481. doi: 10.3389/fmicb.2015.01481 (PMC4701948; doi:10.3389/fmicb.2015.01481)
Supplement: Supplementary file 1 [file DataSheet1.zip › Supplementary material/Supplementary materials and methods.pdf]

Microbial metabolism shifts towards an adverse profile with supplementary iron in the TIM-2 *in vitro* model of the human colon

Guus AM. Kortman, Bas E. Dutilh, Annet JH. Maathuis, Udo F. Engelke, Jos Boekhorst, Kevin P. Keegan, Fiona Nielsen, Jason Betley, Jacqueline Weir, Zoya Kingsbury, Leo AJ. Kluijtmans, Dorine W. Swinkels, Koen Venema, Harold Tjalsma.

Iron quantification in the TIM-2 lumen

Iron content of luminal samples was determined by a ferrozine based assay (Iron Reagent, Liquid FerroZine® Method Kit, ThermoScientific). To determine total iron in the lumen, luminal samples were first treated with acid-potassium permanganate to free the iron. Lumen samples were 2x diluted with MilliQ water (MQ) and were 1:1 mixed with freshly prepared iron releasing reagent (1.2 Mol/L HCl:4.5% $KMnO_4$ (1:1)), within a fume hood. Samples were mixed and incubated for 1h at 60˚C, then 1/8 part ascorbic acid (20% w/v) was added to convert all iron to the Fe^{2+} state. After another incubation for 1h at 60˚C samples were cooled to RT and spun for 5 min at 16,100g to pellet stool particles. 32 µl sample was applied to a microplate (in duplicate) and 160 µl reagent A (kit) was added. After a 2 min incubation the absorbance (A) at 560 nm was recorded with a microplate reader. Next 6.4 µl reagent B (kit) was added and samples were incubated for 10 min at 37˚C. Absorbance (B) was recorded at 560 nm and was corrected for absorbance A. The iron content was calculated by comparing the absorbance with a series of ferrous sulfate standards.

DNA extraction from luminal samples for microarray analysis and high throughput sequencing

200mg of material was initially mixed with 250 µL lysis buffer (AGOWA, mag Mini DNA Isolation Kit, Germany), 250 µL zirconiumbeads (0.1 mm; Biospec Products, USA), and 200 µL phenol, before being introduced to a BeadBeater (BioSpec Products) for two times 2 min. DNA was extracted with the AGOWA mag Mini DNA Isolation Kit and yield was quantified using a NanoDrop ND-1000 spectrophotometer (NanoDrop Technologies) (Crielaard et al., 2011).

Real-time PCR to determine the total number of bacteria in the TIM-2 lumen

DNA extracted from the lumen at 0h and 72h was subjected to quantitative PCR to investigate the variation in the total amount of bacteria after different iron exposures. Quantification was performed using the universal primers 16S-uni-II-F (TCCTACGGGAGGCAGCAGT) and 16S-uni-II-R (GGACTACCAGGGTATCTAATCCTGTT), and probe 16S-uni-II (6FAM-CGTATTAC CGCGGCTGCTGGCAC-TAMRA) (Applied Biosystems, The Netherlands). The amplification was performed with 5 µL DNA sample (1:10 diluted) and 25 µL PCR mixture that contained 15 µL 2× FastStart Universal Probe Mastermix (Roche, Mannheim, Germany), 1 µL of each primer (10 µmol/L) and probe (5 µmol/L), and 7 µL MilliQ water. The assay was run on the 7500 Fast Real-Time PCR system (Applied Biosystems) at the following settings: 1 step of 2 min at 50 °C, 1 step of 10 min at 95 °C, 40 cycles of 15 s at 95 °C and 1 min at 60 °C. Dilutions of a control microbiota were used as quantitative standards (5 fg/µL to 5 ng/µL) (Ladirat et al., 2013).

16S rDNA pyrosequencing of the gut microbiome

16S rDNA pyrosequencing was the main method for studying the gut microbiome composition at the 24h and 72h time points. For the preparation of the amplicon pool for pyrosequencing, the following universal primers were applied for amplification of the V3-V6 region of the 16S rRNA gene: a) forward primer , 5'-*CCATCTCATCCCTGCGTGTCTCCGACTAG*NNNNNN**ACTCCTACGGGAGGCAGCAG**-3' (the

italicized sequence is 454 Life Sciences primer A, and the bold sequence is the broadly conserved bacterial primer 338F; NNNNNN designates the sample-specific six-base barcode used to tag each PCR product); b) reverse primer 5'-*CCTATCCCCTGTGTGCCTTGGCAGTCTCAG***CRRCACGAGCTGACGAC**-3' (the italicized sequence is 454 Life Sciences primer B, and the bold sequence is the broadly conserved bacterial primer 1061R). PCR amplification mixture contained : 1 μL fecal DNA, 1 μL bar-coded forward primer, 15 μL master mix (1 μL KOD Hot Start DNA Polymerase (1 U/μL; Novagen, Madison, WI, USA), 5 μL KOD-buffer (10×), 3 μL MgSO4 (25 mM), 5 μL dNTP mix (2 mM each), 1 μL (10 μM) reverse primer) and 33 μL sterile water (total volume 50 μL). PCR conditions were: 95°C for 2 minutes followed by 35 cycles of 95°C for 20 s, 55°C for 10 s, and 70°C for 15 s. The approximately 750 bp PCR amplicon was purified using the MSB Spin PCRapace kit (Invitek) and subsequently by using the Purelink PCR Purification kit (Invitrogen), with high-cutoff binding buffer B3. DNA concentration of the purified products was checked with a Nanodrop 1000 spectrophotometer (Thermo Scientific). A composite sample for pyrosequencing was prepared by pooling 200 ng of each purified sample and submitted for pyrosequencing of the V3-V4 region of the 16S rRNA gene on the 454 Life Sciences GS-FLX+ platform using Titanium sequencing chemistry (GATC-Biotech, Germany).

Microarray analysis of the microbiota by I-Chip

Analysis of the microbiota with the Intestinal (I)-Chip was executed as described by Maathuis et al. (Maathuis et al., 2012). Briefly, the 'intestinal chip' (I-Chip) has been developed as a fast method to determine the composition of the microbiota. 16S rDNA based sequences of roughly 400 microorganisms have been placed on a DNA micro-array as previously described in detail (Crielaard et al., 2011). The DNA was labeled and hybridized to the microarray. After washing the arrays were scanned and analyzed. Analysis of the composition of the microbiota (using I-Chip) indicated the bacterial genera which were selectively stimulated or suppressed by the iron interventions. Changes in the composition of the microbiota in the experiments were analyzed for the 0, 24, 48 and 72h time points as described below. This analysis was included to validate pyrosequencing results, to study additional time points and to study the effect of iron on low-abundant taxa that were below the limit of detection of 16S rDNA pyrosequencing.

Metagenomic sequencing

DNA of the lowFe and 50FeS conditions at the 72h time point was barcoded, the samples pooled, and sequenced using paired 251 bp sequencing on two lanes of an Illumina HiSeq2500 instrument. The pooled samples were applied to both lanes to minimize potential lane-specific biases.

Cell line, media and growth conditions

The colon adenocarcinoma cell line Caco-2 (obtained from the American Type Culture Collection) was cultured under standard conditions (37°C and 5% CO_2) in DMEM (Lonza) supplemented with 10% fetal calf serum (Invitrogen), 20 mmol/L HEPES, 100 nmol/L nonessential amino acids (Invitrogen), 2 mmol/L L-glutamine (Lonza) and Penicillin/Streptomycin (100 U/mL and 100 µg/mL, respectively) (Pen Strep, Invitrogen). The cells were subcultured every 6 days and used between passage numbers 3-16.

Fecal water (dialysate) cytotoxicity to Caco-2 monolayer assays

Caco-2 cells were allowed to grow and differentiate in 22 days to a polarized tight monolayer on the membrane of a Transwell® Permeable Support (24 wells, 6.5 mm insert) with 0.4 µm polycarbonate membrane (Corning) under standard culture conditions.

Supplementary materials and methods

Dialysis liquid samples (not containing bacterial metabolites) and dialysate samples of the 72h time point were spun for 10 min at 36,000g and 4˚C. Concentrated tannic acid (Riedel-de Haën) was added to the supernatants in a final concentration of 10 µmol/L to precipitate the excess iron. After a 10 min incubation at RT the samples were spun again and the supernatants were filter sterilized by the use of a 0.2 µm filter. Iron removal was confirmed by the ferrozine based assay as described above (without acid-potassium permanganate treatment). Pre-treated dialysates were warmed to 37˚C and 200 µl was applied in triplicate to the apical side of the Caco-2 monolayers after a wash in Hank's Balanced Salt Solution (HBSS; Invitrogen, Cat. 14025-09). As a control, standard maintaining buffer HBSS with 10 µmol/L tannic acid was used. Basolateral compartments were filled with 0.6 ml HBSS. To check for the monolayer integrity, the Trans Epithelial Electrical Resistance (TEER) was periodically measured with the use of the Millicell®-ERS (Millipore). After incubation the basolateral compartments were sampled for lactate dehydrogenase (LDH) quantification and were replaced with 0.5 ml fresh HBSS. Apical compartments were washed once with HBSS and replaced with 0.2 ml filter sterilized 1 mmol/L phenol red in HBSS. The cells were incubated for 1 h at standard conditions, the TEER was determined and the lower compartment was sampled to quantify phenol red permeability. Next, the cells were washed twice with PBS and the basolateral compartment once. To quantify cell death 50 µl 0.4% Trypan blue in PBS was applied to the cells and incubated for 2 min at RT. Then, cells (twice) and basolateral chamber (once) were washed with PBS. We want to emphasize that we removed the iron from the dialysate prior to Caco-2 exposure and this way we only examined the effects of the microbial metabolome and excluded potential additional effects towards the cells due to redox activity of iron. We note that the presence of iron may augment the toxic effects of the metabolome as it has previously been shown that iron can have detrimental effects on the intestinal epithelium (Ferruzza et al., 2003;Natoli et al., 2009).

Phenol red permeability:

Samples from the basolateral compartments were split in technical duplicates of 180 µl and 20 µl 0.1 mol/L NaOH was added. Absorbance was recorded in a microplate reader at 540 nm. The amount of phenol red that was diffused through the monolayers was compared relatively to the control.

LDH quantification:

To investigate the detrimental effect of the dialysates to the Caco-2 monolayers, the LDH release into the basolateral medium was determined. Samples were collected as described and were used in the Cytotox 96® Non-Radioactive Cytotoxicity Assay (Promega) according to the manufacturer's protocol. The LDH concentrations were compared relatively to the control.

Trypan blue-exclusion test:

Washed filters were excised from the insert by scalpel and transferred to a microtube. 200 µl acetone-0.5% (w/v) Na_2SO_4 (7:3) was added and tubes were sonicated in a water bath to extract the trypan blue from the filters. Tubes were spun for 5 min at 16,100g and the supernatant was transferred to a microplate. Absorbance was recorded at 560 nm with 450 nm as reference wavelength. Trypan blue intensity was compared relatively to the control. Adapted from Sakai *et al.* (Sakai et al., 1998).

<u>Statistics and data presentation</u>

Analysis of 16S rDNA sequencing data

Pyrosequencing data were analyzed with a workflow based on QIIME v1.2 (Caporaso et al., 2010) using settings as recommended in the QIIME 1.2 tutorial, with the following exceptions: reads were

filtered for chimeric sequences using Chimera Slayer (Haas et al., 2011). OTU clustering was performed with settings as recommended in the QIIME newsletter of December 17th 2010 (http://qiime.wordpress.com/2010/12/17/new-default-parameters-for-uclust-otu-pickers/) using an identity threshold of 97%. Diversity metrics were calculated as implemented in QIIME 1.2. The specific measured used are mentioned in the figure and figure legends where appropriate. Hierarchical clustering of samples was performed using UPGMA with weighted UniFrac as a distance measure as implemented in QIIME 1.2. The RDP classifier version 2.2 was performed for taxonomic classification, using the default confidence threshold of 0.8 (Cole et al., 2009). Analysis of microbiota composition was done on relative abundance, except for alpha- and beta diversity, which were done on rarified data , using default settings (the average of 5 rarefaction runs). Multivariate redundancy analysis (RDA) was done using Canoco 5.0 (Ter Braak and Smilauer, 2012), using default settings of the analysis type "Constrained-supplementary". For the permutation tests, 500 randomizations were done, keeping duplicate samples as blocks. No relative abundance threshold was used. Details on the underlying mathematics can be found in (Ter Braak and Smilauer, 2012). Visualization of differences in relative abundance of taxa between different study groups was done in Cytoscape (Shannon et al., 2003). Statistical analysis of the pyrosequencing data was done with SciPy (www.scipy.org). Differences in relative abundance between groups at a single time point (cross-sectional) were compared by Mann-Whitney U (MWU) testing. Correlations of taxa with metabolite levels in 24h and 72h samples were assessed by Spearman's correlation rank test. Linear regression (GraphPad Prism version 5.03) was used to plot the best-fit line with 95% confidence interval and to determine whether the deviation of the slope was significantly different from zero (when the deviation was not different from zero the correlation was considered not relevant). Medians of phylogenetic diversity and distances were compared by Kruskal-Wallis test with Dunn's post test, or MWU test (GraphPad Prism version 5.03). The effects of iron on the microbiome were compared among the single conditions, but also as pools as described above. P-values for the microbiome data were not corrected for multiple testing as they were analyzed in an explorative manner.

Analysis of microarray (I-Chip) data

Duplicate measurements at four time points (t=0, 24h, 48h, and 72h) from all incubation conditions were analyzed. Intensity measurements of the I-Chip were normalized (normalized - $\log_2(x+1)$) and scaled between 0 and 1 across all measurements. Next, we calculated Spearman correlations per probe across the time series between all measurements, and calculated permutation p-values by randomizing the measurements for each probe.

Analysis of metagenomic sequencing data

After demultiplexing the metagenomic sequencing reads, stringent Q30 quality trimming was applied using Trimgalore v0.2.4 (www.bioinformatics.babraham.ac.uk/projects/trim_galore/), resulting in 69.9 and 59.8 million quality trimmed read pairs for the lowFe and 50FeS conditions respectively, at an average single ended read length of 207 ± 47 nt. Metagenomic sequencing reads were annotated by aligning them to the human gut gene catalog (Qin et al., 2010) with Bowtie using default parameters (Langmead et al., 2009). This resulted in an alignment rate of 29.0% and 36.8% for the lowFe and 50FeS conditions respectively. The abundances of KEGG orthologs (Kanehisa et al., 2014) and SEED subsystem annotations (level-1 subsystems and the more detailed level-2 subsystems annotation) (Overbeek et al., 2005) were normalized by sample size. Enrichment was calculated as the log2 ratio of the average abundance of the function in the iron datasets over the lowFe datasets, plus a small pseudocount of 10^{-9}. Significance was calculated using a two-sided t-test with unequal variance across all the sequenced datasets. P-values for the metagenome data were not corrected for multiple testing as they were analyzed in an explorative manner.

References

Caporaso, J.G., Kuczynski, J., Stombaugh, J., Bittinger, K., Bushman, F.D., Costello, E.K., Fierer, N., Pena, A.G., Goodrich, J.K., Gordon, J.I., Huttley, G.A., Kelley, S.T., Knights, D., Koenig, J.E., Ley, R.E., Lozupone, C.A., Mcdonald, D., Muegge, B.D., Pirrung, M., Reeder, J., Sevinsky, J.R., Turnbaugh, P.J., Walters, W.A., Widmann, J., Yatsunenko, T., Zaneveld, J., and Knight, R. (2010). QIIME allows analysis of high-throughput community sequencing data. *Nat Methods* 7**,** 335-336.

Cole, J.R., Wang, Q., Cardenas, E., Fish, J., Chai, B., Farris, R.J., Kulam-Syed-Mohideen, A.S., Mcgarrell, D.M., Marsh, T., Garrity, G.M., and Tiedje, J.M. (2009). The Ribosomal Database Project: improved alignments and new tools for rRNA analysis. *Nucleic Acids Res* 37**,** D141-145.

Crielaard, W., Zaura, E., Schuller, A.A., Huse, S.M., Montijn, R.C., and Keijser, B.J. (2011). Exploring the oral microbiota of children at various developmental stages of their dentition in the relation to their oral health. *BMC Med Genomics* 4**,** 22.

Ferruzza, S., Scarino, M.L., Gambling, L., Natella, F., and Sambuy, Y. (2003). Biphasic effect of iron on human intestinal Caco-2 cells: early effect on tight junction permeability with delayed onset of oxidative cytotoxic damage. *Cell Mol Biol (Noisy-le-grand)* 49**,** 89-99.

Haas, B.J., Gevers, D., Earl, A.M., Feldgarden, M., Ward, D.V., Giannoukos, G., Ciulla, D., Tabbaa, D., Highlander, S.K., Sodergren, E., Methe, B., Desantis, T.Z., Human Microbiome, C., Petrosino, J.F., Knight, R., and Birren, B.W. (2011). Chimeric 16S rRNA sequence formation and detection in Sanger and 454-pyrosequenced PCR amplicons. *Genome Res* 21**,** 494-504.

Kanehisa, M., Goto, S., Sato, Y., Kawashima, M., Furumichi, M., and Tanabe, M. (2014). Data, information, knowledge and principle: back to metabolism in KEGG. *Nucleic Acids Res* 42**,** D199-205.

Ladirat, S.E., Schols, H.A., Nauta, A., Schoterman, M.H., Keijser, B.J., Montijn, R.C., Gruppen, H., and Schuren, F.H. (2013). High-throughput analysis of the impact of antibiotics on the human intestinal microbiota composition. *J Microbiol Methods* 92**,** 387-397.

Langmead, B., Trapnell, C., Pop, M., and Salzberg, S.L. (2009). Ultrafast and memory-efficient alignment of short DNA sequences to the human genome. *Genome Biol* 10**,** R25.

Maathuis, A.J., Van Den Heuvel, E.G., Schoterman, M.H., and Venema, K. (2012). Galacto-oligosaccharides have prebiotic activity in a dynamic in vitro colon model using a (13)C-labeling technique. *J Nutr* 142**,** 1205-1212.

Natoli, M., Felsani, A., Ferruzza, S., Sambuy, Y., Canali, R., and Scarino, M.L. (2009). Mechanisms of defence from Fe(II) toxicity in human intestinal Caco-2 cells. *Toxicol In Vitro* 23**,** 1510-1515.

Overbeek, R., Begley, T., Butler, R.M., Choudhuri, J.V., Chuang, H.Y., Cohoon, M., De Crecy-Lagard, V., Diaz, N., Disz, T., Edwards, R., Fonstein, M., Frank, E.D., Gerdes, S., Glass, E.M., Goesmann, A., Hanson, A., Iwata-Reuyl, D., Jensen, R., Jamshidi, N., Krause, L., Kubal, M., Larsen, N., Linke, B., Mchardy, A.C., Meyer, F., Neuweger, H., Olsen, G., Olson, R., Osterman, A., Portnoy, V., Pusch, G.D., Rodionov, D.A., Ruckert, C., Steiner, J., Stevens, R., Thiele, I., Vassieva, O., Ye, Y., Zagnitko, O., and Vonstein, V. (2005). The subsystems approach to genome annotation and its use in the project to annotate 1000 genomes. *Nucleic Acids Res* 33**,** 5691-5702.

Qin, J., Li, R., Raes, J., Arumugam, M., Burgdorf, K.S., Manichanh, C., Nielsen, T., Pons, N., Levenez, F., Yamada, T., Mende, D.R., Li, J., Xu, J., Li, S., Li, D., Cao, J., Wang, B., Liang, H., Zheng, H., Xie, Y., Tap, J., Lepage, P., Bertalan, M., Batto, J.M., Hansen, T., Le Paslier, D., Linneberg, A., Nielsen, H.B., Pelletier, E., Renault, P., Sicheritz-Ponten, T., Turner, K., Zhu, H., Yu, C., Li, S., Jian, M., Zhou, Y., Li, Y., Zhang, X., Li, S., Qin, N., Yang, H., Wang, J., Brunak, S., Dore, J., Guarner, F., Kristiansen, K., Pedersen, O., Parkhill, J., Weissenbach, J., Bork, P., Ehrlich, S.D., and Wang, J. (2010). A human gut microbial gene catalogue established by metagenomic sequencing. *Nature* 464**,** 59-65.

Sakai, M., Imai, T., Ohtake, H., and Otagiri, M. (1998). Cytotoxicity of absorption enhancers in Caco-2 cell monolayers. *J Pharm Pharmacol* 50**,** 1101-1108.

Shannon, P., Markiel, A., Ozier, O., Baliga, N.S., Wang, J.T., Ramage, D., Amin, N., Schwikowski, B., and Ideker, T. (2003). Cytoscape: a software environment for integrated models of biomolecular interaction networks. *Genome Res* 13**,** 2498-2504.

Ter Braak, C.J.F., and Smilauer, P. (2012). *Canoco reference manual and user's guide: software for ordination, version 5.0.* Ithaca, USA: Microcomputer Power.
