# Supplementary material for: Microbial Metabolism Shifts Towards an Adverse Profile with Supplementary Iron in the TIM-2 In vitro Model of the Human Colon
Source: Front Microbiol. 2016 Jan 6;6:1481. doi: 10.3389/fmicb.2015.01481 (PMC4701948; doi:10.3389/fmicb.2015.01481)
Supplement: Supplementary file 1 [file DataSheet1.zip › Supplementary material/Supplementary Table 3.pdf]

Microbial metabolism shifts towards an adverse profile with supplementary iron in the TIM-2 *in vitro* model of the human colon

Guus AM. Kortman, Bas E. Dutilh, Annet JH. Maathuis, Udo F. Engelke, Jos Boekhorst, Kevin P. Keegan, Fiona Nielsen, Jason Betley, Jacqueline Weir, Zoya Kingsbury, Leo AJ. Kluijtmans[1], Dorine W. Swinkels, Koen Venema, Harold Tjalsma.

Supplementary Table 3. ^{1}H-NMR resonances from metabolites observed in dialysate samples at 72h (measured at pH=2.50)

Metabolite	CS [splitting] (ppm)			
Acetate	2.08 [si]			
Alanine	1.50 [do]	3.87 [qu]		
Butyrate	0.91 [tr]	1.60 [sx]	2.35 [tr]	
Cholate†	0.71 [si]			
Ethanol	1.17 [tr]	3.65 [qu]		
Formate	8.24 [si]			
Isobutyrate	1.14 [do]	2.60 [mu]		
Isoleucine	0.94 [tr]	1.02 [do]		
Isovalerate	0.94 [do]	2.02 [mu]	2.26 [do]	
Lactate	1.41 [do]	4.36 [qu]		
Leucine	0.95 [tr]	0.97 [do]		
Methanol	3.35 [si]			
Phenylalanine	7.35 [mu]			
Phenylpropionate	2.71 [tr]	2.94 [tr]	7.32 [mu]	
Propionate	1.06 [tr]	2.20 [qu]		
Succinate	2.66 [si]			
Trimethylamine	2.89 [do]			
Tyrosine	6.89 [do]	7.19 [do]		
Valerate	0.88[tr]	1.32 [mu]	1.56 [mu]	2.32 [tr]
Valine	0.99 [do]	1.05 [do]	2.28 [mu]	3.62 [do]
X1	2.66 [tr]	2.86 [tr]		
X2	1.74 [mu]	3.28 [mu]		
X3	3.55 [tr]			
X4	3.60 [tr]			
X5	6.84 [do]	7.17 [do]		
X6	2.44 [tr]			

† Tentative assignment based on Jacobs *et al*. (Jacobs et al., 2008)
X1-X6 could not with certainty or tentatively be assigned to a known metabolite

References

Jacobs, D.M., Deltimple, N., Van Velzen, E., Van Dorsten, F.A., Bingham, M., Vaughan, E.E., and Van Duynhoven, J. (2008). (1)H NMR metabolite profiling of feces as a tool to assess the impact of nutrition on the human microbiome. *NMR Biomed* 21**,** 615-626.
